# Vapors of Volatile Plant-Derived Products Significantly Affect the Results of Antimicrobial, Antioxidative and Cytotoxicity Microplate-Based Assays

**DOI:** 10.3390/molecules25246004

**Published:** 2020-12-18

**Authors:** Marketa Houdkova, Genesis Albarico, Ivo Doskocil, Jan Tauchen, Klara Urbanova, Edgardo E. Tulin, Ladislav Kokoska

**Affiliations:** 1 Department of Crop Sciences and Agroforestry, Faculty of Tropical AgriSciences, Czech University of Life Sciences Prague, 165 00 Prague, Czech Republic; houdkovam@ftz.czu.cz (M.H.); albarico@ftz.czu.cz (G.A.); 2 Department of Microbiology, Nutrition and Dietetics, Faculty of Agrobiology, Food and Natural Resources, Czech University of Life Sciences Prague, 165 00 Prague, Czech Republic; doskocil@af.czu.cz; 3 Department of Food Science, Faculty of Agrobiology, Food and Natural Resources, Czech University of Life Sciences Prague, 165 00 Prague, Czech Republic; tauchen@af.czu.cz; 4 Department of Sustainable Technologies, Faculty of Tropical AgriSciences, Czech University of Life Sciences Prague, 165 00 Prague, Czech Republic; urbanovak@ftz.czu.cz; 5 Philrootcrops, Visayas State University, Baybay City 6521, Philippines; edgardo.tulin@vsu.edu.ph

**Keywords:** bioassay, broth microdilution, DPPH, essential oil, microtiter plate, MTT, plant compounds, supercritical CO_2_ extract, volatilization

## Abstract

Volatile plant-derived products were observed to exhibit broad spectrum of biological effects. However, due to their volatility, results of conventional microplate-based bioassays can be significantly affected by the vapors. With aim to demonstrate this phenomenon, antimicrobial, antioxidant, and cytotoxic activities of three essential oils (*Alpinia elegans*, *Cinnamomum iners*, and *Xanthostemon verdugonianus*), one supercritical CO_2_ extract (*Nigella sativa*), and four plant-derived compounds (capsaicin, caryophyllene oxide, 8-hydroxyquinoline, and thymoquinone) were evaluated in series of experiments including both ethylene vinyl acetate (EVA) Capmat sealed and nonsealed microplates. The results clearly illustrate that vapor transition to adjoining wells causes false-positive results of bioassays performed in nonsealed microtiter plates. The microplate layout and a duration of the assay were demonstrated as the key aspects defining level of the results affection by the vapors of volatile agents. Additionally, we reported biological activities and chemical composition of essential oils from *A. elegans* seeds and *X. verdugonianus* leaves, which were, according to our best knowledge, analyzed for the first time. Considering our findings, certain modifications of conventional microplate-based assays are necessary (e.g., using EVA Capmat as vapor barrier) to obtain reliable results when biological properties of volatile agents are evaluated.

## 1. Introduction

Volatile plant-derived products (VPDPs) are a large group of carbon-based chemicals with low molecular weight and high vapor pressure at ambient temperature including different chemical classes such as hydrocarbons and their derivatives, e.g., benzoquinones, epoxides, methoxyphenols, and quinolines. [1,2]. Volatile products that can be obtained from different plant parts involve essential oils, extracts, oleoresins, tinctures, distillates, and juice concentrates. They are isolated using an array of techniques such as expression, distillation, concentration, solvent extraction, and supercritical fluid extraction [3]. Essential oils (EOs), the complex mixtures composed mainly of terpenoids, are important representatives of VPDPs with a characteristic aroma and a flavor typical for certain plant families (e.g., Lauraceae, Myrtaceae, and Zingiberaceae) [4,5,6,7]. Since ancient times, EOs have been used for their medicinal and organoleptic properties. Nowadays, plant volatiles have various applications in pharmaceutical, agronomic, food, sanitary, cosmetic, and perfume industries [8]. For example, EO and supercritical CO_2_ extract obtained from seeds of *Nigella sativa* L. is used as a medicament for a variety of disorders in the digestive tract, kidney, cardiovascular, respiratory, and immune systems [9,10]. VPDPs were observed to exhibit broad spectrum of biological effects including antimicrobial, anticarcinogenic, antioxidant, and cytotoxic properties [11]. Especially plant species originating from tropical regions are considered valuable sources of biologically active agents due to the stronger pressure of bacterial and fungal pathogens affecting plants in tropical ecosystems [12]. Among the tropical areas, Philippine archipelago belongs to the important centers of biodiversity with a large number of endemic plants. Besides species that has been reported to exhibit medicinal properties [13], less explored medicinal and aromatic plants, such as *Alpinia elegans* (C.Presl) K.Schum., *Cinnamomum iners* Reinw. ex Blume, and *Xanthostemon verdugonianus* Náves ex Fern.-Vill, occur it this region.

In a plant-based drug discovery, the in vitro biological screening using pharmacologically relevant microplate assays is one of the first steps to verify the effectivity and safety of medicinal plants and their constituents [14]. Since the microwell plate was created in 1951 by Hungarian scientist with aim to provide a potentially useful techniques suitable for the high throughput screening, a number of standardized procedures have been established such as methods widely referenced to Clinical and Laboratory Standards Institute (CLSI) [15]. The use of microplate together with a fully automated equipment makes bioassays simple, fast, and reliable, providing reproducible results [16]. In natural product research, they serve to determine biological effects such as antimicrobial [17], antioxidant [18], and cytotoxic activity [19]. Respective, broth microdilution, 2,2-diphenyl-1-picrylhydrazyl (DPPH) radical scavenging, and thiazolyl blue tetrazolium bromide (MTT) assays are examples of the most widely used methods for the assessment of plant-derived compounds including volatile agents [20,21,22].

Although conventional microplate-based bioassays are common in the laboratory practice, in case of VPDPs in vitro testing, they face specific problems due to physicochemical properties of these agents such as high volatility, hydrophobicity, and viscosity [23]. The hydrophobic nature worsens the solubility of VPDPs in water-based media (e.g., agar and broth), that may reduce the dilution capability and unequal distribution of active components through the medium [24]. The volatility causes a risk of active substance losses by evaporation during sample handling, experiment preparation, and incubation depending on its time and temperature conditions [25,26]. Moreover, significant influence of vapors of volatiles on the results of biological tests performed in microtiter plates by spreading of volatiles into adjoining wells has been described [27]. To prevent above mentioned difficulties, some modifications of standardized methods are required. For example, the use of ethylene vinyl acetate (EVA) Capmat was observed to be effective as a vapor barrier in assays for determination of antistaphylococcal activity of thymoquinone in combinations with antibiotics [28] and cytotoxicity of carvacrol, cinnamaldehyde, eugenol, 8-hydroxyquinoline, thymol, and thymoquinone [29]. However, certain current studies still continue to overlook the significant risk of results affection by vapors of plant volatiles when tested in microplate-based assays [30,31].

With aim to clearly demonstrate the significant results distortion of the standard methods for evaluation of biological properties of VPDPs by their vapors, the series of tests comparing identical experiments performed simultaneously in both sealed and nonsealed microtiter plates were assayed. For this purpose, we tested antimicrobial, antioxidant, and cytotoxic effects of three EOs obtained from Philippine plant species *A. elegans*, *C. iners*, and *X. verdugonianus*, one supercritical CO_2_ extract from *N. sativa*, and four plant-derived compounds, i.e., capsaicin, caryophyllene oxide, 8-hydroxyquinoline, and thymoquinone, as representatives of various classes of biologically effective agents with different levels of volatility. Moreover, chemical composition of EOs and supercritical CO_2_ extract tested was analyzed to assess the relationship between their biological activities and chemistry.

## 2. Results

### 2.1. Antimicrobial Assay

**Figure 1:**
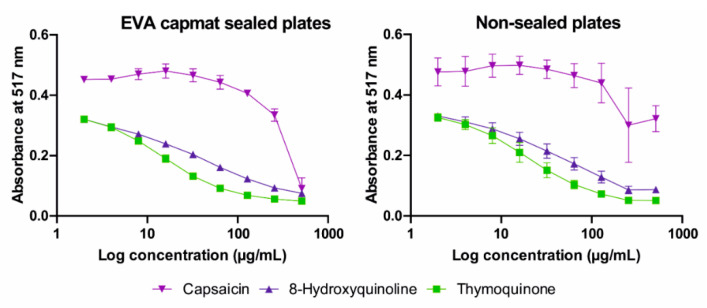
The 2,2-diphenyl-1-picrylhydrazyl radical scavenging activity of capsaicin, 8-hydroxyquinoline, and thymoquinone tested in microtiter plates sealed with vapor barrier ethylene vinyl acetate (EVA) Capmat and nonsealed microplates.

The results of antimicrobial activity performed using multiplate design when all samples were tested in one replicate in one microtiter plate (Figure 1 were significantly affected by vapors of plant-derived products tested in nonsealed plates. In general, the effectiveness of samples varied ranging from 2 to 1024 µg/mL and from 2 to 512 µg/mL in EVA Capmat sealed and nonsealed plates, respectively. Importantly, 8-Hydroxyquinoline was determined as the most active antimicrobial agent, when its lowest minimum inhibitory concentration (MIC) was found against *Staphylococcus aureus* with value 2 µg/mL in both EVA Capmat sealed and nonsealed plates. The most affected result of antimicrobial assay was observed for capsaicin against *Candida albicans*, although no activity was detected in plates sealed with vapor barrier, MIC 64 µg/mL was found in nonsealed plates. Similarly, *A. elegans* oil, *X. verdugonianus* oil, caryophyllene oxide, and thymoquinone did not possessed any growth-inhibitory effect against one of these pathogens *C. albicans*, *Enterococcus faecalis*, and *S. aureus* in EVA Capmat sealed plates; however, certain degree of inhibition with MICs in the range of 256–512 µg/mL was observed in plates without vapor barrier. Except *C. iners* EO, all samples exhibited some antimicrobial efficacy; however, only 8-hydroxyquinoline was active against all pathogens tested. The detailed results of growth-inhibitory effect of VPDPs against four representatives of both Gram-negative and Gram-positive bacteria and one fungal strain in EVA Capmat sealed and nonsealed plates are summarized in Table 1.

### 2.2. Antioxidant Assay

**Figure 2:**
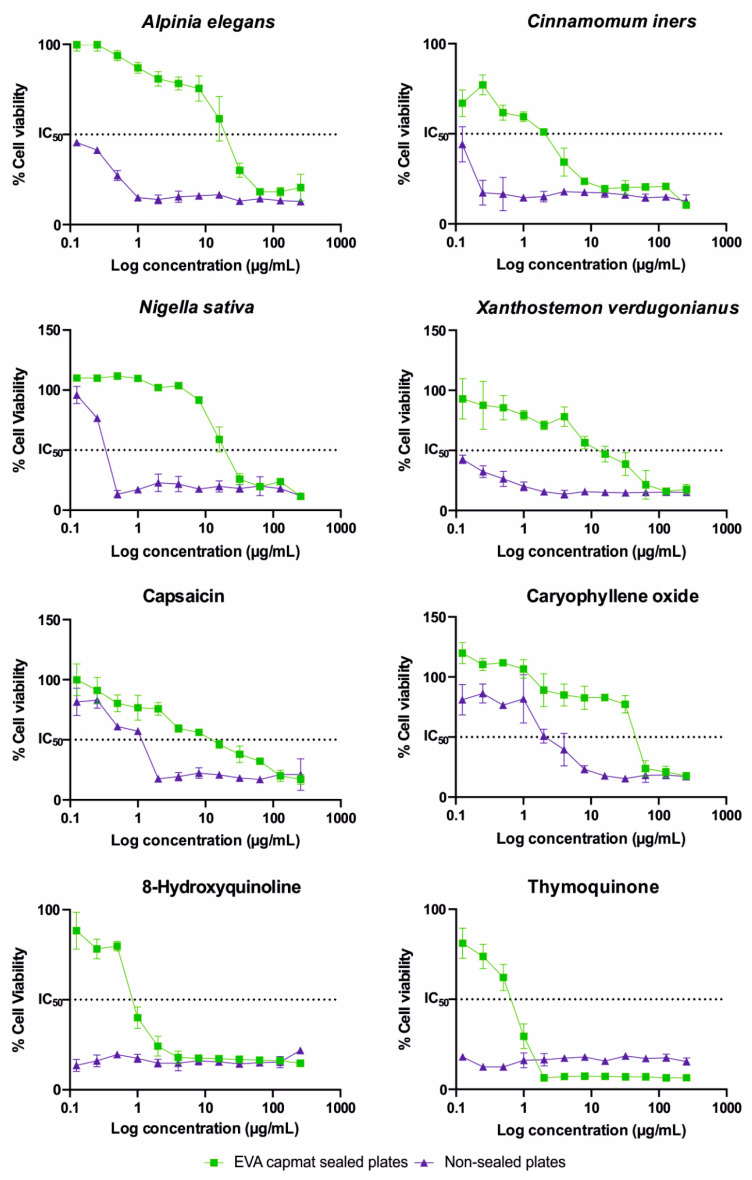
Cytotoxic activity of *Alpinia elegans*, *Cinnamomum iners*, *Xanthostemon verdugonianus* essential oils, *Nigella sativa* supercritical CO_2_ extract, capsaicin, caryophyllene oxide, 8-hydroxyquinoline, and thymoquinone to human colon cancer cells Caco-2 tested in microtiter plates sealed with vapor barrier EVA Capmat and nonsealed microplates.

Although the results of antioxidant assay were affected less than those of antimicrobial testing, VPDPs showed different results in series of single-plate designed DPPH tests when one EO (or extract) and one compound were assayed in triplicates together in the same microtiter plate (Figure 2). Among all plant-derived volatiles, only three compounds, namely, capsaicin, 8-hydroxyquinoline, and thymoquinone, showed some level of antioxidant activity in both EVA Capmat sealed and nonsealed plates with respective half maximal inhibitory concentrations (IC_50_) in the ranges of 24.22–313.36 and 23.11–199.33 µg/mL, respectively. A summary of all results of DPPH assay is shown in Table 2. The most promising free radical scavenging potential has been observed for capsaicin (IC_50_ 24.22 and 23.11 µg/mL in sealed and nonsealed plates, respectively). The result of antioxidant activity of thymoquinone was the most affected by vapors, in contrast to IC_50_ value 313.36 µg/mL in EVA Capmat sealed plates, lower value 199.33 µg/mL was detected in nonsealed plates. In addition, certain level of vapors influence is apparent regarding to standard deviations of IC_50_ values, which are represented by broader range of values in nonsealed plates as seen in Figure 1 showing data of absorbance average of triplicates in one experiment.

### 2.3. Cytotoxicity Assay

Similarly, as in both antimicrobial and antioxidant assays, the results of cytotoxicity were significantly affected when tested in single-plates layouts with four samples in duplicates in one microtiter plate (Figure 2. The values IC_50_ varied in ranges of 0.95–57.40 and 0.18–4.85 µg/mL for EVA Capmat sealed and nonsealed microplates, respectively. The detailed results of the MTT assay performed with human colon cancer cells Caco-2 are listed in Table 3. The lowest cytotoxic effect was observed for caryophyllene oxide (IC_50_ value 57.40 µg/mL) in EVA Capmat sealed plates. Moreover, in case of this compound, the most significant difference in the results was recorded as IC_50_ value determined in plates with vapor barrier was 11 times higher than IC_50_ value in nonsealed plates (IC_50_ = 4.85 µg/mL). The effect of vapors of volatile agents tested on results of cytotoxic assay is obvious when graph curves for each sample tested is compared as shown in Figure 2 displaying data from three independent experiments in duplicates. Moreover, IC_50_ values of *A. elegans* oil, *C. iners* oil, *X. verdugonianus* oil, 8-hydroxyquinoline, and thymoquinone performed in nonsealed plates were not detected, as these values were below the lowest concentration tested.

### 2.4. Gas Chromatography/Mass Spectrometry (GC/MS) Analysis

In this study, three EOs hydrodistilled from different parts of Philippines plant species *A. elegans*, *C. iners*, and *X. verdugonianus* were obtained in yields ranging from 0.52% to 2.86% (*v*/*w*). Yield of supercritical CO_2_ extract of *N. sativa* was 5.80% (*w*/*w*). Based on the GC/MS analysis equipped with HP-5MS/DB-HeavyWAX columns, a total of 119, 106, 51, and 20 compounds were identified in the samples, representing 93.637/93.186, 95.571/96.676, 94.757/96.114, and 82.349/92.308% of their total contents, respectively. The analysis showed that monoterpenes and sesquiterpenes were the leading chemical classes of the major constituents in the EOs tested, however, *N. sativa* supercritical CO_2_ extract was composed mainly by fatty acids. The complete chemical composition of all VPDPs analyzed is provided in Table 4, Table 5, Table 6 and Table 7.

In *A. elegans* seed EO, D-limonene (16.770/15.394% = 2.390/2.333 mg/kg) was the main compound followed by α-pinene (13.661/12.237% = 1.963/1.855 mg/kg) and caryophyllene oxide (11.368/10.781% = 1.738/1.772 mg/kg). *C. iners* leaf EO was rich in content of caryophyllene (21.002/34.875% = 3.223/6.561 mg/kg), followed by linalool (15.466/13.899% = 3.153/3.023 mg/kg). Pseudolimonene was detected in a significant amount by HP-5MS column (9.549% = 1.715 mg/kg), and, conversely, β-phellandrene was found by DB-HeavyWAX column (5.982% = 1.080 mg/kg). The major component of *X. verdugonianus* leaf oil was α-gurjunene (32.285/19.519% = 3.741/3.648 mg/kg), followed by cyperenone (22.653/52.694% = 2.745/10.958 mg/kg) and caryophyllene (6.386/2.987% = 0.739/0.559 mg/kg). The most abundant component of *N. sativa* supercritical CO_2_ extract was linoleic acid (71.657/59.245% = 3.019/6.713), followed by ethyl linoleate (5.023/1.582% = 0.138/0.174 mg/kg) and ethyl oleate (2.782/0.265% = 0.072/0.030 mg/kg). Other dominant compounds, oleic acid (19.576% = 2.208 mg/kg) and hexadecenoic acid (9.897% = 1.097 mg/kg), were detected by DB-HeavyWAX column only.

## 3. Discussion

Based on the presented results, this study clearly demonstrates that the vapors of VPDPs can significantly affect results of microplate-based bioassays, as shown by the differences between the values observed for the plates sealed with vapor barrier and nonsealed plates covered with their lid only. Similar phenomenon has previously been observed in experiments assaying antistaphylococcal, toxic, and antifungal potentials of volatile agents such as thymoquinone, phenol, and plant EOs [28,34,35]. According to these findings, it is apparent that the results of assays evaluating biological activities of volatile agents in nonsealed microtiter plates might be unreliable. Unsealed wells are exposed to losses of bioactive compounds by evaporation, which can cause false-negative results [36]. On the other hand, vapors transition to adjoined wells can produce false-positive results of the tests [27], which is evident especially when the multiplate design of experiments is used. For this reason, the microplate layout is the important aspect affecting the accuracy of the results in nonsealed experiments. Considering the plate layouts, the results of the antimicrobial assay showed that the samples situated in rows closer to the most active volatile agents (8-hydroxyquinoline and thymoquinone) possessed lower or no growth-inhibitory effect in plates sealed with vapor barrier in comparison to nonsealed plates. Similarly, in MTT assay, the samples tested in the wells next to 8-hydroxyquinoline or thymoquinone were evaluated to be so highly toxic that their IC_50_ values were not detected in nonsealed microplates as these values were below the lowest concentration tested. The same case occurred in our previous study [29] when high toxicity of carvacrol, eugenol, and thymol was observed in nonsealed experiments, whereas nontoxic potential of these compounds was determined in EVA Capmat sealed plates. Moreover, single-plate layouts with samples tested in one replicate in one microtiter plate are also affected by vapor losses of active agents and their transition to adjoining wells. Beside the design of microplate layout, a duration of the assay is a crucial parameter affecting the results of assessment of biological potential of volatiles. In the study on antistaphylococcal effect of thymoquinone, the increasing concentration of this compound during 5 h was detected by GC/MS in the microplate wells that were initially thymoquinone free [27]. Therefore, in the case of short-term tests such as DPPH assay, the influence of the vapors did not occur to such an extent because its incubation lasts only 30 min, in contrast to respective incubation times 24 and 72 h of standard antimicrobial and cytotoxicity assays.

As far as biological activity of VPDPs tested in this study is considered, *N. sativa* seed supercritical CO_2_ extract is only one previously assessed for its antimicrobial effect. It exhibited growth-inhibitory activity with MIC values range of 16–128 µg/mL against standard strains of *C. albicans*, *E. faecalis*, *Escherichia coli*, *Pseudomonas aeruginosa*, and *S. aureus* [37]. In the case of *A. elegans*, the result can be supported by our previous study on *A. elegans* leaf EO where MIC value 512 µg/mL was determined against *S. aureus* [38]. Only weak antimicrobial potential of *C. iners* leaf methanol extract against *C. albicans*, *E. coli*, *P. aeruginosa*, and *S aureus* was described by Mustafa et al. [39] with MIC values ranging from 780 to 25,000 µg/mL. Data on antimicrobial activity of *X. verdugonianus* are completely missing, nevertheless recent study concerting related plant species *X. youngii* from Thailand determined its antistaphylococcal activity with MIC value 1250 µg/mL [40]. Growth-inhibitory effects of capsaicin, caryophyllene oxide, 8-hydroxyquinoline, and thymoquinone were evaluated against various *S. aureus* strains by several authors with respective MIC values >50, 60, 4, and 16 µg/mL [41,42,43,44], which are corresponding to our results in nonsealed plates.

There is a lack of data on antioxidant potential of EOs obtained from the abovementioned plant species with the exception of *C. iners* leaf oil that was observed to possess antioxidant effect with IC_50_ value of 218.88 µg/mL [45]. Other study has previously reported DPPH radical scavenging activity of ethanol extract from *A. elegans* leaves with IC_50_ value of 97.58 µg/mL [46]. However, EOs of these both species did not produce any activity in our study. In case of *N. sativa* supercritical CO_2_ extract, our result might be considered as similar to Solati et al. [47] who observed a low level of antioxidant activity of this extract with IC_50_ value of 2590 µg/mL. In general, the results of DPPH assay in our study evaluating antioxidant activity of volatile compounds are in correspondence with those obtained by other authors except Karakaya et al. [48] who detected antioxidant effect of caryophyllene oxide with IC_50_ value of 84.09 µg/mL, whereas this compound did not possess any activity in our study. Similar to the results presented here, Nascimento et al. [49] showed high radical scavenging potential of capsaicin expressed as half maximal effective concentration with value of 23.1 µg/mL and Cherdtrakulkiat et al. [50] demonstrated 8-hydroxyquinoline to exhibit antioxidant effect with IC_50_ value of 89.24 µg/mL. However, Yildiz et al. [51] recorded relatively very high IC_50_ value for thymoquinone (about 800,000 µg/mL).

There is no previous literature reporting any cytotoxic effect for EOs isolated from *A. elegans* seeds, *C. iners* leaves, and *X. verdugonianus* leaves tested here, however, EOs obtained from another plant parts or their extracts have been reported. In case of *A. elegans*, toxicity of leaf oil was assessed against human lung cells in our previous study (IC_50_ = 27.7 µg/mL) [38]. According to the results of Mustafa et al. [52] who evaluated acute toxicity of *C. iners* leaf methanol extract using a brine shrimp assay, this plant species is considered safe, although high toxic potential was found in our study for *C. iners* seed EO. In case of *N. sativa* supercritical CO_2_ extract, a medium cytotoxic effect was observed against human breast cancer cells with IC_50_ value of 53.34 µg/mL [53]. Numerous assays for testing the cytotoxicity of plant-derived compounds have previously been performed on various human cancer cell lines from tissues such as a bone marrow, a colon epithelium, and a peripheral blood. Similar to our results, all compounds tested here, i.e., capsaicin, caryophyllene oxide, 8-hydroxyquinoline, and thymoquinone, have been observed to possess a certain degree of the cytotoxicity with respective IC_50_ values of 18.3, 57.7, 1.3, and 3.0–8.0 µg/mL [54,55,56,57].

The biological properties of EOs and supercritical CO_2_ extract tested in this study have been attributed to their chemical composition primarily rich in monoterpenes, sesquiterpenes, and fatty acids. The chemical profile of *C. iners* leaf oil and *N. sativa* seed supercritical CO_2_ extract has previously been described, whereas literature about chemical analysis of EOs from *A. elegans* seeds and *X. verdugonianus* leaves is not available. When comparing analytical data in this study with previously published works on *C. iners* leaf oil, its chemical composition corresponds to results of Son et al. [58], who detected β-caryophyllene, caryophyllene oxide, and humulene as the main components. Although, *N. sativa* seeds are known for their high content of thymoquinone, as described, e.g., in study of Venkatachallam et al. [59], the supercritical CO_2_ extract analyzed in our study was observed to contain a high level of linoleic acid and other fatty acids. This finding was confirmed by [47,60] who also detected dominant prevalence of linoleic acid (60.74%) and low amount of thymoquinone (0.28–1.42%). In our previous study [38], we identified caryophyllene oxide (24.70/30.50%), α-pinene (9.70/10.50%), isolongifolol methyl ether (4.20/3.80%), and linalool (4.10%) as major compounds of *A. elegans* leaf oil, which resembles chemical profile of EO obtained from its seeds. In addition to determination of raw percentages of peak areas, concentration of components in 1 kg of dry plant material was computed using predicted relative response factors with aim to increase the accuracy and reliability of the volatile compounds’ quantification. This approach is important in technological processes with several applications in the field of chemical analysis of VPDPs as it enables the quantification of volatile compounds by GC/MS with flame-ionization detection without having authentic compounds available, and also, it can avoid time-consuming calibration procedures [61].

## 4. Materials and Methods

### 4.1. Chemicals and Reagents

With aim to evaluate agents of different volatility characterized by distinct values of a vapor pressure, following plant-derived compounds: capsaicin (95%, CAS 404-86-4, 1.32 × 10^−8^ mm Hg at 25 °C), caryophyllene oxide (99%, CAS 1139-30-6, 7.00 × 10^−3^ mm Hg at 25 °C), 8-hydroxyquinoline (99%, CAS 148-24-3, 1.66 × 10^−3^ mm Hg at 25 °C), and thymoquinone (99%, CAS 490-91-5, 6.00 × 10^−2^ mm Hg at 25 °C) were assayed. Ciprofloxacin (98%, CAS 85721-33-1), fluconazole (98%, CAS 86386-73-4), oxacillin (86.3%, CAS 7240-38-2), and tetracycline (98–102%, CAS 60-54-8) were used as positive antibiotic controls. Other chemicals used were as follows: DPPH (CAS 1898-66-4), *n*-hexane (CAS 110-54-3), 6-hydroxy-2,5,7,8-tetramethylchromane-2-carboxylic acid (Trolox, CAS 53188-07-1), dimethyl sulfoxide (DMSO, CAS 67-68-5), methanol (CAS 67-56-1), MTT (CAS 298-93-1), and Tween 20% (CAS 9005-64-5). α-Bisabolol (CAS 23089-26-1), camphene (CAS 79-92-5), carvone (CAS 6485-40-1), caryophyllene (CAS 87-44-5), geraniol (CAS 106-24-1), humulene (CAS 6753-98-6), linalool (CAS 126-91-0), methyl octanoate (CAS 111-11-5), myrcene (CAS 123-35-3), α-pinene (CAS 7785-70-8), β-pinene (CAS 18172-67-3), α-terpinene (CAS 99-86-5), γ-terpinene (CAS 99-85-4), and terpinolene (CAS 586-62-9) were used as analytical standards. With exception, methanol and DMSO purchased from Penta (Prague, Czech) and *n*-hexane from Merck KGaA (Darmstadt, Germany), all other chemicals were obtained from Sigma-Aldrich (Prague, Czech).

### 4.2. Plant Material

The seeds of *A. elegans* and leaves of *C. iners* and *X. verdugonianus* were collected in the foothills of Mount Pangasugan located on the island Leyte (Philippines) in April 2018. The seeds of *N. sativa* were purchased in local spice store U Salvatora (Prague, CZ). The plants were authenticated by ethnobotanist Ladislav Kokoska from the Department of Tropical Crop Sciences and Agroforestry, the Faculty of Tropical AgriSciences, Czech University of Life Sciences (CZU), Prague (CZ), and by taxonomist Edwino S. Fernando from the Institute of Biology Jose Vera Santos Memorial Herbarium, College of Science, University of the Philippines, Diliman (PHL). The voucher specimens of *A. elegans*, *C. iners*, and *X. verdugonianus* and voucher sample of *N. sativa* seeds were deposited in the herbarium of the Department of Botany and Plant Physiology of the Faculty of Agrobiology, Food and Natural Resources, CZU Prague (CZ). Dried plant material was ground and homogenized by Grindomix apparatus (GM 100 Retsch, Haan, Germany). The residual moisture content was evaluated gravimetrically at 130 °C by Scaltec SMO 01 analyzer (Scaltec Instruments, Gottingen, Germany) in triplicates. A detailed botanical description and physicochemical characteristic of plant samples including scientific name, family, voucher specimen/sample number, area of collection, part used, isolation technique for obtaining of EOs and supercritical CO_2_ extract, their yield, and color are summarized in Table 8.

### 4.3. Hydrodistillation

Essential oils were obtained from *A. elegans*, *C. iners*, and *X. verdugonianus* by hydrodistillation of ground dried plant material in 1 L of distilled water for 3 h using a Clevenger-type apparatus (Merci, Brno, Czech) according to the procedures described in the European Pharmacopoeia [62]. The essential oils were stored in sealed glass vials at 4 °C. The data on yields (v/w, based on the dry plant weight) of obtained essential oils are shown in Table 8.

### 4.4. Supercritical Fluid Extraction

Supercritical CO_2_ extraction of *N. sativa* seeds was carried out using Speed SFE Helix system (Applied Separations, Allentown, PA, USA). Initially, 10 g of ground material were filled into the 100 mL stainless steel extraction vessel between two layers of glass wool and subsequently installed into the extraction module. The extraction process was than performed using following parameters: isocratic pressure 200 Ba, temperature 40 °C and CO_2_ flow rate 5 LPM. The extracts were stored in sealed glass vials at 4 °C. The properties and yield (*w*/*w*, based on the dry plant weight) of obtained extracts are shown in Table 8.

### 4.5. Bacterial Strains and Culture Media

The following four bacterial and one yeast standard strains of the American Type Culture Collection (ATCC) were used: *C. albicans* ATCC 90028, *E. faecalis* ATCC 29212, *E. coli* ATCC 25922, *P. aeruginosa* ATCC 27853, and *S. aureus* ATCC 29213. All strains were purchased from Oxoid (Basingstoke, UK). Cation-adjusted Mueller-Hinton broth (MHB) (Oxoid) equilibrated to pH 7.6 with a Trizma base (Sigma-Aldrich) was used as the cultivation and assay medium for all bacteria tested, whereas further supplementation by 1% of glucose (Sigma Aldrich) was done in case of *E. faecalis*.

Stock cultures of bacterial strains were cultivated in broth medium at 37 °C for 24 h prior to testing. For the preparation of inoculum, the turbidity of the bacterial suspension was adjusted to 0.5 McFarland standard using a Densi-La-Meter II (Lachema, Brno, Czech) to obtain a final concentration of 10^8^ CFU/mL.

### 4.6. Cell Cultures

Human colon cancer cells Caco-2 obtained from ATCC (Rockville, MD, USA) were propagated in Eagle’s Minimum Essential Medium (EMEM) obtained from Biowest (Nuaille, FR) supplemented with 10% fetal bovine serum, 1% sodium bicarbonate, 1% sodium pyruvate, 5 mM glutamine, 1% Minimum Essential Medium nonessential amino acids, and 1% penicillin-streptomycin solution (10,000 units/mL of penicillin and 10 mg/mL of streptomycin). The components for cells’ cultivation were purchased from Sigma-Aldrich. Cultures were incubated at 37 °C in a humidified atmosphere of 5% CO_2_ in the air.

### 4.7. Antimicrobial Assay

**Figure 3:**
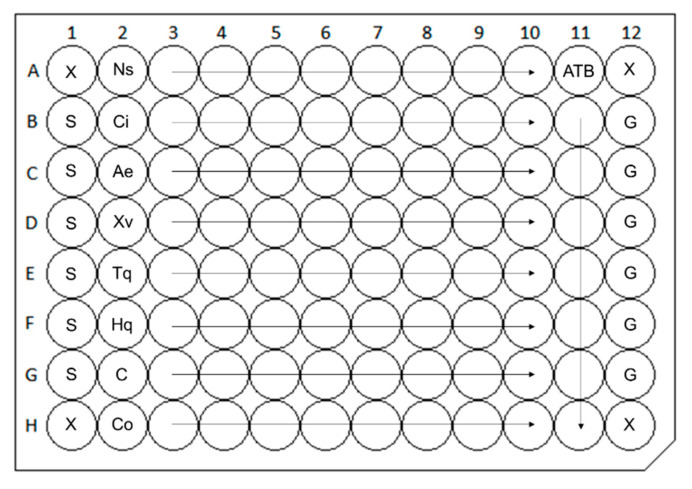
Scheme of multiplate design of broth microdilution assay. Ns: *Nigella sativa*, Ci: *Cinnamomum iners*, Ae: *Alpinia elegans*, Xv: *Xanthostemon verdugonianus*, Tq: thymoquinone, Hq: hydroxyquinoline, C: capsaicin, Co: caryophyllene oxide—nine serial twofold dilutions of volatile agents tested, ATB: eight serial twofold dilutions of positive antibiotic control, G: growth control (inoculated broth, 100% growth of bacteria), S: sterility control (noninfected medium control, 0% growth of bacteria), X: empty wells (not used in data calculation).

The in vitro antibacterial potential of EOs, supercritical CO_2_ extract, and volatile compounds was determined using a broth microdilution method according to the guidelines of the CLSI [63]. Each sample of volatile agents was dissolved in DMSO and diluted in MHB in a range of 2–1024 μg/mL using an automated pipetting platform Freedom EVO 100 equipped with a four-channel liquid handling arm (Tecan, Mannedorf, Switzerland). Plates were inoculated with bacterial suspension and incubated at 37 °C for 24 h sealed/nonsealed with vapor barrier EVA Capmat (Micronic, Aston, PA, USA). Bacterial growth was measured spectrophotometrically using a Multimode Reader Cytation 3 (BioTek Instruments, Winooski, VT, USA) at 405 nm. The MICs were determined as the lowest concentrations that inhibited bacterial growth by ≥80% compared with that of the agent-free growth control and expressed in microgram per milliliter. DMSO assayed as the negative control did not inhibit any of the strains tested. The susceptibilities of *C. albicans*, *P. aeruginosa*, and *S. aureus*, to fluconazole, ciprofloxacin, and oxacillin, respectively, and susceptibilities of *E. faecalis* and *E. coli* to tetracycline were checked as positive antibiotic controls [64]. All experiments were carried out in triplicate in three independent experiments and the results were expressed as median/modal MIC values. The multiplate design of broth microdilution assay when eight different samples were tested in one microtiter plate is described in Figure 3.

### 4.8. Antioxidant Assay

**Figure 4:**
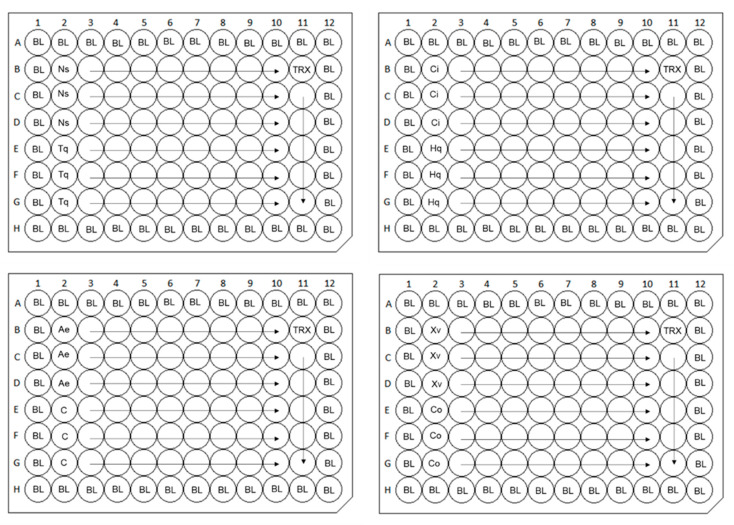
Scheme of single-plate designs with triplicates of two samples in one microtiter plate for DPPH assay. Ns: *Nigella sativa*, Tq: thymoquinone, Ci: *Cinnamomum iners*, Hq: 8-hydroxyquinoline, Ae: *Alpinia elegans*, C: capsaicin, Xv: *Xanthostemon verdugonianus*, Co: caryophyllene oxide—nine serial twofold dilutions of volatile agents tested; BL: blank control (pure methanol, 0% of radical inhibition); TRX: six twofold dilutions of positive Trolox control.

The DPPH radical scavenging assay was performed using a slightly modified method previously described by Sharma and Bhat [65]. Initially, EOs, supercritical CO_2_ extract, and plant-derived compounds were dissolved in DMSO and diluted in methanol to obtain concentration of 1024 μg/mL. Subsequently, serial dilutions of each sample were prepared in absolute methanol (100 μL) in 96-well microtiter plates using the automated pipetting platform Freedom EVO 100. Trolox was used as a standard reference material and pure methanol as blank control. The radical-antioxidant reaction was started after adding 75 μL of absolute methanol and 25 μL of freshly prepared 1 mM DPPH in methanol to each well, creating a range of concentrations from 0.25 to 512 μg/mL (final volume of 200 μL). The plates were kept in the dark at room temperature for 30 min nonsealed/sealed with vapor barrier EVA Capmat. Absorbance was measured at 517 nm using Cytation 3 microplate reader. All tests were performed in triplicates at three independent experiments. Results were expressed as IC_50_ with standard deviation (±SD) in microgram per milliliter. The single-plate design of DPPH assay when two samples in triplicates are tested in one microplate is presented in Figure 4.

### 4.9. Cytotoxicity Assay

**Figure 5:**
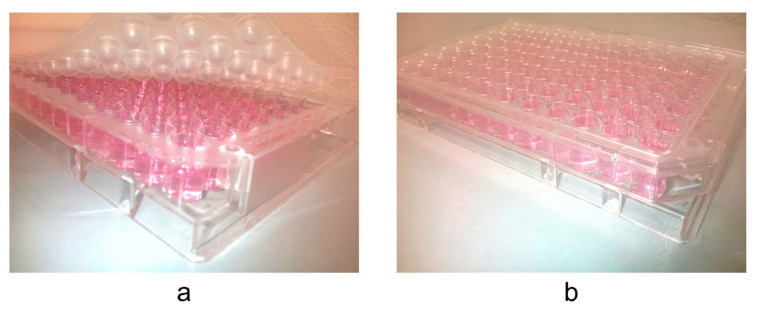
Thiazolyl blue tetrazolium bromide cytotoxicity assay performed in (**a**) the microtiter plate sealed with vapor barrier EVA Capmat and (**b**) nonsealed microtiter plate covered with the lid only.

**Figure 6:**
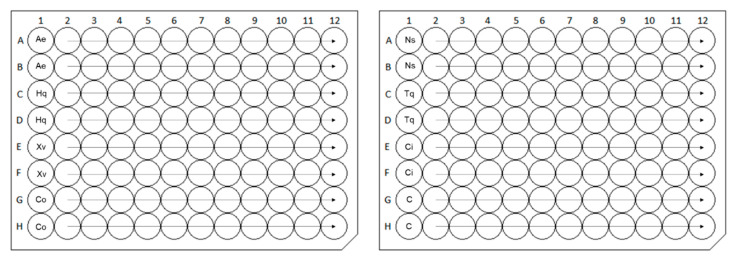
Scheme of single-plate designs with duplicates of four samples in one microtiter plate for thiazolyl blue tetrazolium bromide cytotoxicity assay. Ae: *Alpinia elegans*, Hq: 8-hydroxyquinoline, Xv: *Xanthostemon verdugonianus*, Co: caryophyllene oxide, Ns: *Nigella sativa*, Tq: thymoquinone, Ci: *Cinnamomum iners*, C: capsaicin—12 serial twofold dilutions of volatile agents tested.

Cell viability was measured using a modified MTT cytotoxicity assay originally developed by Mosmann [66]. Caco-2 cell lines were seeded in 96-well plates at a density of 2.5 × 10^3^ cells per well. After 24 h, the cells were treated with twofold serially diluted samples (0.25–512 μg/mL) of EOs, supercritical CO_2_ extract, and compounds dissolved in DMSO and cultivated for 72 h with/without vapor barrier EVA Capmat (Figure 5). Thereafter, MTT reagent (1 mg/mL) in EMEM solution was added to each well and the plates were incubated for an additional 2 h at 37 °C in a humidified atmosphere of 5% CO_2_ in the air. The media with MTT were removed and the intracellular formazan product was dissolved in 100 μL of DMSO. The solvent used did not affect the viability of the intestinal cells. The absorbance was measured at 555 nm using a Tecan Infinite M200 spectrometer (Tecan Group, Mannedorf, Switzerland), and the viability was calculated in comparison to an untreated control. Three independent experiments (two replicates each) were performed for every test. The single-plate design when four different samples in duplicates are tested in one microtiter plate is shown in Figure 6. The results of the cytotoxicity effect were calculated by GraphPad Prism software (GraphPad Software, Inc., La Jolla, CA, USA) and expressed as average IC_50_ value with standard deviation in microgram per milliliter. The levels of cytotoxic effects were classified according to the Special Program for Research and Training in Tropical Diseases (WHO—Tropical Diseases) [67] as cytotoxic (IC_50_ < 2 μg/mL), moderately cytotoxic (IC_50_ 2–89 μg/mL), and nontoxic (IC_50_ > 90 μg/mL).

### 4.10. GC/MS Analysis

For determination of the main components of EOs and supercritical CO_2_ extract, GC/MS analysis was performed using the dual-column/dual-detector gas chromatograph Agilent GC-7890B system equipped with autosampler Agilent 7693, two columns, a fused-silica HP-5MS column (30 m × 0.25 mm, film thickness 0.25 μm, Agilent 19091s-433) and a DB-HeavyWAX (30 m × 0.25 mm, film thickness 0.25 μm, Agilent 122–7132), and a flame ionization detector (FID) coupled with single quadrupole mass selective detector Agilent MSD-5977B (Agilent Technologies, Santa Clara, CA, USA). Operational parameters were as follows: helium as a carrier gas at 1 mL/min and injector temperature 250 °C for the both columns. The oven temperature was raised for the both columns after 3 min from 50 to 280 °C. Initially, after an isothermic period of 3 min, the heating rate was 3 °C/min until the temperature reached 120 °C. Subsequently, the heating velocity increased to 5 °C/min until it reached 250 °C, and after 5 min of holding time on 250 °C, the heating rate increased to 15 °C/min until it reached 280 °C. Heating was followed by the isothermic period of 20 min. The essential oils were diluted in *n*-hexane for GC/MS at a concentration of 20 µg/mL, and for a quantitative analysis, 1 μL of methyl octanoate was added as an internal standard. Precisely, 1 μL of each EO solution was injected in a split mode (split ratio 1:50). The mass detector was set to the following conditions: ionization energy 70 eV, ion source temperature 230 °C, scan time 1 s, and mass range 40–600 *m*/*z*.

Identification of the constituents was based on the comparison of their retention indices, retention times and spectra with the National Institute of Standards and Technology Library ver. 2.0.f (NIST, USA) [32], as well as with authentic standards (Sigma-Aldrich) and literature [33]. The RI were calculated for compounds separated by H5-5MS column using the retention times of *n*-alkanes series ranging from C8 to C40 (Sigma-Aldrich). For each EO and supercritical CO_2_ extract analyzed, the final number of compounds was calculated as the sum of components simultaneously identified using the both columns and the remaining constituents identified by individual column only. Quantitative data were computed as described in Cachet et al. [68] using the following formula:

$$m_{i}=RRF_{i}^{Pred}m_{MO}\frac{A_{i}}{A_{MO}},$$

where $m_{i}$ is the mass of the compound i to be quantified, expressed in milligram per 1 kg of the plant dry weight (DWP); $RRF_{i}^{Pred}$ predicted relative response factor of compound i, $m_{MO}$ mass of methyl octanoate (internal standard, IS), $A_{i}$ and $A_{MO}$ are the peak areas of the analyte and the IS, respectively, determined by the FID. Moreover, relative percentage contents of identified components have been determined using the FID data and indicated for the both columns.

## 5. Conclusions

The results of experiments presented in this study clearly demonstrate that the vapors of VPDPs can significantly affect the results of standard microplate-based bioassays. In series of experiments using EVA Capmat sealed and nonsealed microplates, antimicrobial, antioxidant, and cytotoxic activities of three EOs from Philippine less explored plant species (*A. elegans*, *C. iners*, and *X. verdugonianus*), one supercritical CO_2_ extract from *N. sativa* and four plant compounds (capsaicin, caryophyllene oxide, 8-hydroxyquinoline, and thymoquinone) were evaluated. It was confirmed that vapor transition causes false-positive results of the bioassays performed in nonsealed microtiter plates. The microplate layout and a duration of the assay were demonstrated as the crucial aspects defining level of the results affection by the vapors of volatile agents. In several cases, no antimicrobial activity was detected in sealed plates, however, certain grown-inhibitory effect was found in nonsealed plates. As well as in the cytotoxicity assay, significant differences in results were recorded between sealed and nonsealed plates. Due to the strong effect of the vapors of the most cytotoxic agents, toxicity of the samples in adjoining wells was not detected in nonsealed plates. Only capsaicin, 8-hydroxyquinoline, and thymoquinone showed some level of antioxidant activity, while IC_50_ values of thymoquinone were the most affected by vapors. However, in DPPH assay, the influence of the vapors was not occurred to such an extent because this is a short-term test. Additionally, we reported biological activities and chemical composition of EOs from *A. elegans* seeds and *X. verdugonianus* leaves, which were, according to our best knowledge, analyzed for the first time. Due to our findings, certain modifications of the conventional bioassays performed in microtiter plates are necessary for evaluation of biological properties of the volatile agents (e.g., using of vapor barrier) in order to protect against vapor transition and to obtain reliable results.

## Figures and Tables

**Table 1:** Influence of the vapors of volatile plant-derived products on the results of the antibacterial activity when tested by broth microdilution assay.

Plant Species/Compound	Bacterium/Minimal Inhibitory Concentration (µg/mL)									
	*Candida albicans*		*Enterococcus faecalis*		*Escherichia coli*		*Pseudomonas aeruginosa*		*Staphylococcus aureus*	
	Sealed	Nonsealed	Sealed	Nonsealed	Sealed	Nonsealed	Sealed	Nonsealed	Sealed	Nonsealed
**Essential oil, CO_2_ extract**										
*Alpinia elegans*	>1024	512	>1024	>1024	>1024	>1024	>1024	>1024	256	256
*Cinnamomum iners*	>1024	>1024	>1024	>1024	>1024	>1024	>1024	>1024	>1024	>1024
*Nigella sativa*	>1024	>1024	>1024	>1024	>1024	>1024	>1024	>1024	512	512
*Xanthostemon verdugonianus*	1024	256	>1024	>1024	>1024	>1024	>1024	>1024	>1024	256
**Compound**										
Capsaicin	>1024	64	>1024	>1024	>1024	>1024	>1024	>1024	>1024	256
Caryophyllene oxide	>1024	256	>1024	>1024	>1024	>1024	>1024	>1024	>1024	>1024
8-Hydroxyquinoline	32	16	512	128	256	64	1024	512	2	2
Thymoquinone	64	32	>1024	512	512	264	>1024	>1024	64	16
**Positive antibiotic control**										
Ciprofloxacin	-	-	-	-	-	-	0.125	1	-	-
Fluconazole	0.5	4	-	-	-	-	-	-	-	-
Oxacillin	-	-	-	-	-	-	-	-	0.5	0.5
Tetracycline	-	-	32	32	1	2	-	-	-	-

**Table 2:** Influence of the vapors of volatile plant-derived products on the results of antioxidant activity testing using 2,2-diphenyl-1-picrylhydrazyl assay.

Plant Species/Compound	IC_50_ ± SD ^1^ (µg/mL)	
	Sealed	Nonsealed
**Essential oil, CO_2_ extract**		
*Alpinia elegans*	>512	>512
*Cinnamomum iners*	>512	>512
*Nigella sativa*	>512	>512
*Xanthostemon verdugonianus*	>512	>512
**Compound**		
Capsaicin	24.22 ± 2.57	23.11 ± 7.10
Caryophyllene oxide	>512	>512
8-Hydroxyquinoline	79.09 ± 24.15	61.92 ± 16.53
Thymoquinone	313.36 ± 68.71	199.33 ± 88.02
**Positive control**		
Trolox	9.94 ± 2.30	10.96 ± 1.96

^1^ IC_50_ ± SD: half maximal inhibitory concentration ± standard deviation.

**Table 3:** Influence of the vapors of volatile plant derived products on the results of cytotoxicity to human colon cancer cells Caco-2 determined using thiazolyl blue tetrazolium bromide (MTT) assay.

Plant Species/Compound	IC_50_ ± SD ^1^ (µg/mL)	
	Sealed	Nonsealed
**Essential oil, CO_2_ extract**		
*Alpinia elegans*	23.84 ± 3.29	n.d.^2^
*Cinnamomum iners*	2.96 ± 0.28	n.d.
*Nigella sativa*	21.71 ± 2.79	0.18 ± 0.04
*Xanthostemon verdugonianus*	12.51 ± 3.62	n.d.
**Compound**		
Capsaicin	11.95 ± 2.72	1.71 ± 0.26
Caryophyllene oxide	57.40 ± 9.19	4.85 ± 1.03
8-Hydroxyquinoline	3.24 ± 1.50	n.d.
Thymoquinone	0.95 ± 0.05	n.d.

^1^ IC_50_ ± SD: half maximal inhibitory concentration of proliferation ± standard deviation, ^2^ n.d.: not detected.

**Table 4:** Chemical composition of *Alpinia elegans* seed essential oil.

	RI ^1^		Compound	C ^2^	RF ^3^	Column ^4^				Identification ^5^	
	Obs.	Lit.				HP-5MS		DB-HeavyWAX		HP-5MS	DB-HeavyWAX
						(%)	c	(%)	c		
1	924	924	α-Thujene	MH	0.765	0.117	0.016	- ^8^	-	RI, GC/MS	-
2	932	932	α-Pinene	MH	0.765	13.661	1.963	12.237	1.855	RI, GC/MS. Std	GC/MS
3	945	953	Camphene	MH	0.765	0.245	0.035	0.225	0.032	RI, GC/MS. Std	GC/MS
4	951	957	2.4(10)-Thujadiene	MH	0.779	0.079	0.011	-	-	RI, GC/MS	-
5	971	975	4(10)-Thujene	MH	0.765	0.282	0.040	-	-	RI, GC/MS	-
6	973	974	β-Pinene	MH	0.765	0.521	0.073	0.455	0.066	RI, GC/MS. Std	GC/MS
7	990	988	Myrcene	SH	0.765	0.433	0.061	0.466	0.071	RI, GC/MS. Std	GC/MS
8	1002	1004	Pseudolimonene	MH	0.765	0.090	0.013	-	-	RI, GC/MS.	-
9	1024	1026	m-Cymene	MH	0.700	1.578	0.203	1.722	0.232	RI, GC/MS	GC/MS
10	- ^6^	1029	β-Phellandrene	MH	-	-	-	1.431	0.208	-	GC/MS
11	1030	1031	D-Limonene	MH	0.765	16.770	2.390	15.394	2.333	RI, GC/MS	GC/MS
12	1089	1098	α-Campholenal	MO	0.887	0.147	0.024	0.630	0.112	RI, GC/MS	GC/MS
13	1097	1095	α-Pinene oxide	MO	0.887	0.146	0.024	0.148	0.026	RI, GC/MS	GC/MS
14	1116	1102	Thujone	MO	0.887	0.028	0.005	-	-	RI, GC/MS	-
15	1121	1123	1R.4R-p-Mentha-2.8-dien-1-ol	MO	0.887	0.342	0.056	0.291	0.047	RI, GC/MS	GC/MS
16	1131	1131	4-Acetyl-1-methylcyclohexene	MO	0.911	0.065	0.011	0.017	0.003	RI, GC/MS	GC/MS
17	-	1131	Limona ketone	MO	-	-	-	0.187	0.034	-	GC/MS
18	1134	1136	Limonene epoxide	MO	0.887	0.036	0.006	0.061	0.011	RI, GC/MS	GC/MS
19	1139	1137	L-Pinocarveol	MO	0.887	0.625	0.102	0.645	0.115	RI, GC/MS	GC/MS
20	-	1142	(*E*)-Limonene oxide	MO	-	-	-	0.189	0.034	-	GC/MS
21	1145	1145	Verbenol	MO	0.887	0.542	0.068	0.492	0.087	RI, GC/MS	GC/MS
22	-	1146	Camphor	MO	-	-	-	0.139	0.023	-	GC/MS
23	1160	1163	3-Pinanone	MO	0.887	0.042	0.007	-	-	RI, GC/MS	-
24	1162	1165	Pinocarvone	MO	0.907	0.080	0.013	0.096	0.017	RI, GC/MS	GC/MS
25	1165	1166	Linderol	MO	0.869	0.056	0.009	-	-	RI, GC/MS	-
26	1177	1177	Terpinen-4-ol	MO	0.869	0.509	0.081	0.635	0.092	RI, GC/MS	GC/MS
27	1186	1183	Cryptone	K	0.911	0.570	0.096	0.450	0.082	RI, GC/MS	GC/MS
28	1191	1186	α-Terpineol	MO	0.869	0.131	0.021	-	-	RI, GC/MS	-
29	-	1195	Myrtenol	MO	-	-	-	0.126	0.022	-	GC/MS
30	-	1195	(Z)-Piperitol	MO	-	-	-	0.076	0.013	-	GC/MS
31	1196	1196	Myrtenal	MO	0.907	0.427	0.071	0.294	0.053	RI, GC/MS	GC/MS
32	1199	1281	(4-Isopropenyl-1-cyclohexen-1-yl)methanol	MO	0.887	0.021	0.003	0.045	0.008	RI, GC/MS	GC/MS
33	1208	1204	Berbenone	MO	0.907	0.198	0.033	-	-	RI, GC/MS	-
34	1220	1229	Carveol	MO	0.887	0.546	0.089	0.103	0.018	RI, GC/MS	GC/MS
35	1229	1231	cis-p-Mentha-1(7).8-dien-2-ol	MO	0.887	0.0451	0.007	0.032	0.006	RI, GC/MS	GC/MS
36	-	1239	Isobornyl formate	MO	-	-	-	0.357	0.072	-	GC/MS
37	1241	1244	2-Methyl-3-phenylpropanal	MO	0.824	0.059	0.009	-	-	RI, GC/MS	-
38	1245	1243	Carvone	MO	0.907	0.697	0.116	0.580	0.106	RI, GC/MS, Std	GC/MS
39	1254	1294	Limonene dioxide	MO	1.019	0.013	0.004	-	-	RI, GC/MS	-
40	1276	1196	3-p-Menthen-7-al	MO	0.887	0.131	0.021	-	-	RI, GC/MS	-
41	1287	1287	Pichtosin	MO	0.957	0.042	0.007	-	-	RI, GC/MS	-
42	1292	1228	D-Verbenone	MO	0.907	0.033	0.005	-	-	RI, GC/MS	-
43	1342	1343	Tricycloekasantalal	A	0.867	0.069	0.011	-	-	RI, GC/MS	-
44	-	1345	α-Cubebene	SH	-	-	-	0.115	0.017	-	GC/MS
45	-	1371	Cyclosativene	SH	-	-	-	0.052	0.008	-	GC/MS
46	1380	1374	α-Copaene	SH	0.751	0.878	0.122	0.514	0.077	RI, GC/MS	GC/MS
47	-	1374	Longicyclene	SO	-	-	-	0.279	0.046	-	GC/MS
48	-	1388	β-Cubebene	SH	-	-	-	0.088	0.013	-	GC/MS
49	1395	1389	β-Elemen	SH	0.751	2.001	0.277	2.223	0.342	RI, GC/MS	GC/MS
50	1413	1409	α-Gurjunene	SH	0.751	0.033	0.005	-	-	RI, GC/MS	-
51	1419	1422	α-Bergamotene	SH	0.751	0.142	0.020	0.045	0.007	RI, GC/MS	GC/MS
52	1424	1424	α-Santalene	SH	0.715	3.154	0.415	1.413	0.213	RI, GC/MS	GC/MS
53	1426	1418	Caryophyllene	SH	0.715	2.972	0.392	3.576	0.550	RI, GC/MS, Std	GC/MS
54	-	1436	γ-Elemene	SH	-	-	-	0.035	0.005	-	GC/MS
55	1448	1443	Guaia-6.9-diene	SH	0.715	0.118	0.016	-	-	RI, GC/MS	-
56	1452	1452	Epi-β-Santalene	MH	0.751	0.425	0.059	0.395	0.060	RI, GC/MS	GC/MS
57	-	1457	Alloaromadendrene	SH	-	-	-	0.041	0.006	-	GC/MS
58	1460	1452	Humulene	SH	0.751	1.198	0.166	0.958	0.144	RI, GC/MS, Std	GC/MS
59	-	1464	epi-β-Caryophyllene	SH	-	-	-	0.082	0.012	-	GC/MS
60	1467	1443	Aromandendrene	SH	0.751	0.070	0.010	-	-	RI, GC/MS	-
61	1481	1478	γ-Muurolene	SH	0.715	0.177	0.023	-	-	RI, GC/MS	-
62	1490	1473	2-Isopropenyl-4*a*.8-dimethyl-1.2.3.4.4*a*.5.6.7-octahydronaphthalene	SH	0.745	2.097	0.288	-	-	RI, GC/MS	-
63	1492	1489	β-Eudesmene	SH	0.756	0.518	0.072	-	-	RI, GC/MS	-
64	1498	1498	Eremophilene	SH	0.751	1.448	0.209	1.301	0.196	RI, GC/MS	GC/MS
65	-	1498	α-Selinene	SH	-	-	-	0.224	0.034	-	GC/MS
66	1506	1475	α-Himachalene	SH	0.751	0.746	0.103	-	-	RI, GC/MS	-
67	1514	1505	β-Bisabolene	SH	0.751	4.270	0.591	4.804	0.738	RI, GC/MS	GC/MS
68	-	1515	Cubebol	SO	-	-	-	0.525	0.086	-	GC/MS
69	-	1522	Calamenene	SH	-	-	-	4.906	0.709	-	GC/MS
70	1526	1522	α-Maaliene	SH	0.751	2.823	0.391	3.115	0.469	RI, GC/MS	GC/MS
71	1531	1521	Calamenene	SH	0.707	4.460	0.581	-	-	RI, GC/MS	-
72	1536	1632	Ledene oxide-(II)	O	0.830	0.195	0.030	-	-	RI, GC/MS	-
73	1539	1370	α-Ylangene	SH	0.751	0.159	0.022	-	-	RI, GC/MS	-
74	-	1544	α-Calacorene	SH	-	-	-	0.103	0.018	-	GC/MS
75	-	1549	Elemol	SO	-	-	-	0.040	0.006	-	GC/MS
76	1550	1562	Cadala-1(10).3.8-triene	SH	0.760	0.405	0.057	-	-	RI, GC/MS	-
77	-	1565	β-Calacorene	SH	-	-	-	0.025	0.004	-	GC/MS
78	1568	1565	Nerolidol	SO	0.819	0.035	0.005	-	-	RI, GC/MS	-
79	-	1576	Spathulenol	SO	-	-	-	0.448	0.076	-	GC/MS
80	1595	1582	Caryophyllene oxide	SO	0.830	11.368	1.738	10.781	1.772	RI, GC/MS	GC/MS
81	1613	1602	Ledol	SO	0.819	0.521	0.075	0.251	0.041	RI, GC/MS	GC/MS
82	1620	1610	Humulene epoxide 2	SO	0.830	2.132	0.326	1.676	0.286	RI, GC/MS	GC/MS
83	1623	1630	α-Acorenol	SO	0.819	0.122	0.018	0.407	0.067	RI, GC/MS	GC/MS
84	1626	1678	Aromadendrene oxide-(2)	SO	0.830	0.795	0.116	0.438	0.073	RI, GC/MS	GC/MS
85	1637	1627	Epicubenol	SO	0.819	0.856	0.027	0.626	0.103	RI, GC/MS	GC/MS
86	-	1640	Caryophylladienol II	SO	-	-	-	0.478	0.081	-	GC/MS
87	-	1646	α-Muurolol	SO	-	-	-	0.243	0.040	-	GC/MS
88	1655	1645	Cubenol	SO	0.819	0.071	0.011	-	-	RI, GC/MS	-
89	-	1662	Allohimachalol	SO	-	-	-	1.210	0.199	-	GC/MS
90	1671	1669	Intermedeol	SO	0.819	1.729	0.261	1.053	0.176	RI, GC/MS	GC/MS
91	-	1675	Ylangenal	SO	-	-	-	0.059	0.010	-	GC/MS
92	1678	1685	α-Bisabolol	SO	0.819	1.383	0.209	1.137	0.196	RI, GC/MS	GC/MS
93	1682	1612	Isoaromadendrene epoxide	SO	0.830	3.813	0.612	0.201	0.025	RI, GC/MS	GC/MS
94	1689	1679	(*E*)-α-Santalal	SO	0.841	1.391	0.233	1.456	0.264	RI, GC/MS	GC/MS
95	1700	1689	Cedr-8-en-13-ol	O	0.830	0.034	0.005	-	-	RI, GC/MS	-
96	1756	1740	Isolongifolol	SO	0.819	0.814	0.123	0.806	0.132	RI, GC/MS	GC/MS
97	-	1766	Costol	SO	-	-	-	0.198	0.033	-	GC/MS
98	1814	1809	Ambrial	SO	0.821	0.908	0.137	0.954	0.160	RI, GC/MS	GC/MS
99	-	1899	Corymbolone	SO	-	-	-	0.042	0.008	-	GC/MS
100	-	- ^7^	Menthen-2-ol	MO	-	-	-	0.123	0.021	-	GC/MS
101	-	-	2-Isopropenyl-4*a*.8-dimethyl-1.2.3.4.4*a*.5.6.7-octahydronaphthalene	SH	-	-	-	2.146	0.323	-	GC/MS
102	-	-	Isopiperitenol	MO	-	-	-	0.100	0.018	-	GC/MS
103	-	-	β-(*Z*)-Curcumen-12-ol	SO	-	-	-	0.106	0.018	-	GC/MS
104	-	-	Germacra-4(15).5.10(14)-trien-1β-ol	SO	-	-	-	0.058	0.010	-	GC/MS
105	-	-	1-Methyl-8-(1-methylethyl)-tricyclo [4.4.0.0(2.7)]dec-3-ene-3-methanol	SO	-	-	-	0.333	0.055	-	GC/MS
106	-	-	Diepicedrene-1-oxide	SO	-	-	-	0.175	0.029	-	GC/MS
107	-	-	2,5,8-Trimethyltetralin	SH	-	-	-	0.273	0.039	-	GC/MS
108	-	-	Neointermedeol	SO	-	-	-	0.360	0.059	-	GC/MS
109	-	-	Epiglobulol	SO	-	-	-	0.709	0.117	-	GC/MS
110	-	-	4-(2.4.4-Trimethyl-cyclohexa-1.5-dienyl)-but-3-en-2-one	MO	-	-	-	0.294	0.052	-	GC/MS
111	-	-	Bicyclo [4.4.0]dec-2-ene-4-ol. 2-methyl-9-(prop-1-en-3-ol-2-yl)-	SO	-	-	-	0.409	0.074	-	GC/MS
112	-	-	(2E)-2-Methyl-4-(2.6.6-trimethyl-1-cyclohexen-1-yl)-2-buten-1-ol	MO	-	-	-	0.697	0.114	-	GC/MS
113	-	-	ent-Germacra-4(15).5.10(14)-trien-1β-ol	SO	-	-	-	1.662	0.284	-	GC/MS
114	-	-	7-Isopropenyl-1.4*a*-dimethyl-4.4*a*.5.6.7.8-hexahydro-3H-naphthalen-2-one	SO	-	-	-	0.096	0.013	-	GC/MS
115	-	-	2.6-Ditert-butyl-4-methylphenyl 1-benzyl-2-methylcyclopropanecarboxylate	MO	-	-	-	0.076	0.012	-	GC/MS
116	-	-	1.1’-Bis(cyclooct-2-en-4-one)	MO	-	-	-	0.112	0.021	-	GC/MS
117	-	-	Methyl hexadeca-7.10.13-trienoate	E	-	-	-	0.070	0.012	-	GC/MS
118	-	-	3-Deoxyestradiol	S	-	-	-	0.424	0.067	-	GC/MS
119	-	-	1-Heptatriacotanol	O	-	-	-	0.117	0.017	-	GC/MS
			**Chemical classes**								
			Aldehydes			0.069		-			
			Ketones			0.570		0.450			
			Esters			-		0.070			
			Monoterpene hydrocarbons			33.768		31.859			
			Oxygenated monoterpenes			4.961		6.545			
			Sesquiterpene hydrocarbons			28.102		26.505			
			Oxygenated sesquiterpenes			25.938		27.216			
			Sterols			-		0.424			
			Others			0.229		0.181			
			Total identified (%)			93.637		93.186			

^1^ Retention indices: Obs = retention indices determined relative to a homologous series of *n*-alkanes (C8-C40) on a HP-5MS column, Lit = literature RI values [32,33]; ^2^ C = chemical class: A—aldehydes, E—esters, K—ketones, MH—monoterpene hydrocarbons, MO—oxygenated monoterpenes, O—others, S—sterols, SH—sesquiterpene hydrocarbons, SO—oxygenated sesquiterpenes; ^3^ RF = response factor; ^4^ column = composition of essential oil detected on HP-5MS and DB-HeavyWAX columns; (%) = relative percentage content; c = content is expressed as concentration in milligram per 1 kg of dry plant material; ^5^ identification method: GC/MS = mass spectrum was identical to that of the National Institute of Standards and Technology Library (ver. 2.0.f), RI = the retention index was matching literature database; Std = constituent identity confirmed by coinjection of authentic standards; ^6^ retention indices were not calculated for compounds identified only by DB-HeavyWAX column; ^7^ literature data not available; ^8^ not detected.

**Table 5:** Chemical composition of *Cinnamomum iners* leaf essential oil.

	RI ^1^		Compound	C ^2^	RF ^3^	Column ^4^				Identification ^5^	
	Obs.	Lit.				HP-5MS		DB-HeavyWAX		HP-5MS	DB-HeavyWAX
						(%)	c	(%)	c		
1	924	924	α-Thujene	MH	0.765	0.075	0.013	0.043	0.008	RI, GC/MS	GC/MS
2	930	932	α-Pinene	MH	0.765	0.369	0.066	0.210	0.049	RI, GC/MS, Std	GC/MS
3	974	974	β-Pinene	MH	0.765	0.056	0.009	- ^8^	-	RI, GC/MS, Std	-
4	990	988	Myrcene	MH	0.765	1.116	0.187	0.679	0.164	RI, GC/MS, Std	GC/MS
5	1003	1002	α-Phellandrene	MH	0.765	1.125	0.202	0.810	0.144	RI, GC/MS	GC/MS
6	1015	1014	α-Terpinene	MH	0.765	0.407	0.074	-	-	RI, GC/MS, Std	-
7	1024	1022	o-Cymene	MH	0.698	1.511	0.245	1.314	0.229	RI, GC/MS	GC/MS
8	- ^6^	1024	D-Limonene	MH	-	-	-	1.479	0.326	-	GC/MS
9	-	1029	β-Phellandrene	MH	-	-	-	5.982	1.080	-	GC/MS
10	1029	1004	Pseudolimonene	MH	0.765	9.549	1.715	-	-	RI, GC/MS	-
11	1048	1044	β-Ocimene	MH	0.765	0.096	0.017	0.089	0.015	RI, GC/MS	GC/MS
12	1058	1054	γ-Terpinene	MH	0.765	0.149	0.025	0.099	0.019	RI, GC/MS, Std	GC/MS
13	1088	1086	Terpinolene	MH	0.765	0.118	0.020	0.350	0.073	RI, GC/MS, Std	GC/MS
14	1105	1095	Linalool	MO	0.869	15.466	3.153	13.899	3.023	RI, GC/MS, Std	GC/MS
15	1122	1121	(*Z*)-2-Menthenol	MO	0.869	0.177	0.031	-	-	RI, GC/MS	-
16	1140	1136	(*E*)-2-Menthenol	MO	0.869	0.114	0.022	-	-	RI, GC/MS	-
17	1178	1174	Terpinen-4-ol	MO	0.869	0.965	0.170	-	-	RI, GC/MS	-
18	1186	1183	Cryptone	MO	0.911	0.303	0.056	-	-	RI, GC/MS	-
19	1191	1186	α-Terpineol	MO	0.869	0.895	0.158	0.729	0.203	RI, GC/MS	GC/MS
20	1196	1195	(Z)-Piperitol	MO	0.869	0.031	0.005	0.082	0.019	RI, GC/MS	GC/MS
21	1202	1143	(Z)-Sabinol	MO	0.887	0.033	0.006	-	-	RI, GC/MS	-
22	1208	1207	(E)-Piperitol	MO	0.869	0.052	0.010	-	-	RI, GC/MS	-
23	1241	1244	2-Methyl-3-phenylpropanal	MO	0.824	0.039	0.007	-	-	RI, GC/MS	-
24	1256	1255	Geraniol	MO	0.869	0.472	0.102	0.580	0.127	RI, GC/MS, Std	GC/MS
25	1276	1273	Phellandral	MO	0.887	0.155	0.028	-	-	RI, GC/MS	-
26	1291	1285	Safrole	MO	0.969	2.028	0.402	1.983	0.486	RI, GC/MS	GC/MS
27	1353	1345	α-Cubebene	SH	0.751	0.032	0.004	-	-	RI, GC/MS	-
28	1362	1356	Eugenol	SO	0.947	0.631	0.122	0.631	0.133	RI, GC/MS	GC/MS
29	1369	1389	Longifolene	SH	0.751	0.086	0.010	-	-	RI, GC/MS	-
30	1380	1374	α-Copaene	SH	0.751	0.452	0.069	0.414	0.082	RI, GC/MS	GC/MS
31	1388	1387	β-Bourbonene	SH	0.751	0.069	0.007	-	-	RI, GC/MS	-
32	1395	1389	β-Elemen	SH	0.751	0.267	0.041	-	-	RI, GC/MS	-
33	-	1403	Methyleugenol	SO	-	-	-	0.079	0.020	-	GC/MS
34	1412	1571	Sesquisabinene hydrate	SO	0.819	0.260	0.042	0.184	0.041	RI, GC/MS	GC/MS
35	-	1414	β-Funebrene	SH	-	-	-	1.223	0.215	-	GC/MS
36	-	1417	α-Santalene	SH	-	-	-	0.935	0.404	-	GC/MS
37	1432	1418	Caryophyllene	SH	0.751	21.002	3.223	34.875	6.561	RI, GC/MS, Std	GC/MS
38	1435	1419	β-Ylangene	SH	0.751	0.183	0.028	-	-	RI, GC/MS	-
39	1440	1439	α-Bergamotene	SH	0.751	0.633	0.097	-	-	RI, GC/MS	-
40	1446	1602	Ledol	SO	0.751	0.143	0.022	0.067	0.015	RI, GC/MS	GC/MS
41	1453	1452	Epi-β-Santalene	MH	0.751	0.112	0.017	0.054	0.012	RI, GC/MS	GC/MS
42	-	1455	Geranyl acetone	SO	-	-	-	0.126	0.030	-	GC/MS
43	1456	1460	4.11.11-Trimethyl-8-methylenebicyclo[7.2.0]undec-3-ene	SO	0.751	0.302	0.046	-	-	RI, GC/MS	-
44	1461	1452	Humulene	SH	0.751	4.902	0.753	2.982	0.536	RI, GC/MS, Std	GC/MS
45	1464	1413	Isocaryophyllene	SH	0.751	0.112	0.017	8.067	1.449	RI, GC/MS	GC/MS
46	1470	1464	2-epi-β-Caryophyllene	SH	0.751	10.216	1.570	-	-	RI, GC/MS	-
47	-	1477	γ-Gurjunene	SH	-	-	-	0.235	0.049	-	GC/MS
48	1479	1472	Cadina-3.9-diene	SH	0.751	0.042	0.009	2.235	0.394	RI, GC/MS	GC/MS
49	-	1480	α-Curcumene	SH	-	-	-	0.162	0.026	-	GC/MS
50	1482	1478	γ-Muurolene	SH	0.751	0.269	0.045	-	-	RI, GC/MS	
51	1487	1484	Germacrene D	SH	0.751	0.827	0.127	0.374	0.063	RI, GC/MS	GC/MS
52	1490	1505	β-Farnesene	SH	0.751	1.161	0.178	0.684	0.144	RI, GC/MS	GC/MS
53	1492	1485	β-Selinene	SH	0.751	0.074	0.013	-	-	RI, GC/MS	-
54	1494	1443	Aromandendrene	SH	0.751	0.095	0.016	-	-	RI, GC/MS	-
55	-	1496	Cadina-1.3.5-triene	SH	-	-	-	0.071	0.014	-	GC/MS
56	1500	1496	Viridiflorene	SH	0.751	0.374	0.052	-	-	RI, GC/MS	-
57	1502	1505	Bicyclogermacren	SH	0.751	0.407	0.068	-	-	RI, GC/MS	-
58	1505	1500	α-Muurolene	SH	0.751	0.410	0.069	0.359	0.065	RI, GC/MS	GC/MS
59	1512	1505	β-Bisabolene	SH	0.751	1.051	0.177	1.137	0.200	RI, GC/MS	GC/MS
60	1515	1512	β-Curcumene	SH	0.751	0.031	0.005	-	-	RI, GC/MS	-
61	-	1518	Myristicin	MO	-	-	-	0.174	0.048	-	GC/MS
62	1520	1513	γ-Cadinene	SH	0.751	0.688	0.106	-	-	RI, GC/MS	-
63	1530	1522	δ-Cadinene	SH	0.751	2.095	0.322	-	-	RI, GC/MS	-
64	1537	1454	α-Patchoulene	SH	0.751	0.043	0.007	-	-	RI, GC/MS	-
65	1539	1535	Cubenene	SH	0.751	0.040	0.007	-	-	RI, GC/MS	-
66	1544	1537	α-Cadinene	SH	0.751	0.099	0.019	0.131	0.023	RI, GC/MS	GC/MS
67	1547	1536	α-Bisabolene	SH	0.751	0.088	0.017	-	-	RI, GC/MS	-
68	1550	1544	α-Calacorene	SH	0.715	0.090	0.013	0.135	0.022	RI, GC/MS	GC/MS
69	1567	1564	Nerolidol	SO	0.819	0.482	0.081	0.330	0.060	RI, GC/MS	GC/MS
70	-	1576	Spathulenol	SO	-	-	-	0.150	0.026	-	GC/MS
71	1579	1570	Caryophyllenyl alcohol	SO	0.819	0.887	0.149	0.827	0.151	RI, GC/MS	GC/MS
72	1587	1576	Spathulenol	SO	0.830	0.663	0.085	0.468	0.085	RI, GC/MS	GC/MS
73	1593	1582	Caryophyllene oxide	SO	0.830	2.080	0.425	2.196	0.431	RI, GC/MS	GC/MS
74	1600	1600	Viridiflorol	SO	0.819	0.127	0.023	0.053	0.012	RI, GC/MS	GC/MS
75	1609	1590	Globulol	SO	0.819	0.561	0.094	0.570	0.105	RI, GC/MS	GC/MS
76	-	1610	Humulol	SO	-	-	-	0.092	0.021	-	GC/MS
77	1614	1614	Tetradecanal	A	0.806	1.168	0.193	0.991	0.180	RI, GC/MS	GC/MS
78	1619	1610	Humulene oxide 2	SO	0.830	0.331	0.056	0.105	0.024	RI, GC/MS	GC/MS
79	1623	1645	Cubenol	SO	0.819	0.021	0.003	0.144	0.032	RI, GC/MS	GC/MS
80	1627	1616	Widdrol	SO	0.819	0.314	0.053	0.311	0.055	RI, GC/MS	GC/MS
81	-	1630	α-Acorenol	SO	-	-	-	0.221	0.039	-	GC/MS
82	1637	1619	1.10-Diepicubenol	SO	0.819	0.114	0.022	0.247	0.047	RI, GC/MS	GC/MS
83	-	1640	α-epi-Muurolol	SO	-	-	-	1.109	0.205	-	GC/MS
84	1647	1628	Caryophylladienol I	SO	0.830	0.538	0.091	0.472	0.114	RI, GC/MS	GC/MS
85	1651	1641	α-Cadinol	SO	0.819	3.417	0.573	2.300	0.418	RI, GC/MS	GC/MS
86	1655	1645	δ-Cadinol	SO	0.819	0.284	0.048	0.231	0.042	RI, GC/MS	GC/MS
87	-	1662	Longifolenaldehyde	SO	-	-	-	0.115	0.021	-	GC/MS
88	1680	1612	Isoaromadendrene epoxide	SO	0.830	0.460	0.078	-	-	RI, GC/MS	-
89	1689	1685	α-Bisabolol	SO	0.819	0.141	0.026	-	-	RI, GC/MS, Std	-
90	1695	1694	(1*R*.7*S*)-Germacra-4(15).5.10(14)-trien-1β-ol	SO	0.830	0.101	0.019	0.318	0.060	RI, GC/MS	GC/MS
91	-	1695	Farnesol	SO	-	-	-	0.075	0.017	-	GC/MS
92	1699	1699	2-Pentadecanone	K	0.799	0.053	0.010	0.047	0.011	RI, GC/MS	GC/MS
93	1725	2201	Geranylgeraniol	SO	0.795	0.057	0.011	-	-	RI, GC/MS	-
94	-	1740	Isolongifolol	SO	-	-	-	0.111	0.026	-	GC/MS
95	1753	1747	1-Bisabolone	SO	0.830	0.051	0.010	-	-	RI, GC/MS	-
96	1770	1717	Cyperenone	SO	0.841	0.097	0.019	-	-	RI, GC/MS	-
97	1795	1798	Hexadec-7-enal	A	0.802	0.083	0.015	-	-	RI, GC/MS	-
98	1846	1845	Hexahydrofarnesyl acetone	SO	0.782	0.240	0.038	-	-	RI, GC/MS	-
99	1880	1877	Hexadec-2-enal	A	0.802	0.087	0.016	-	-	RI, GC/MS	-
100	1895	1903	Homosalate	E	0.935	0.034	0.008	-	-	RI, GC/MS	-
101	1922	1922	Farnesyl acetone	SO	0.806	0.337	0.056	0.316	0.055	RI, GC/MS	GC/MS
102	-	1960	Hexadecanoic acid	FA	-	-	-	0.724	0.132	-	GC/MS
103	1967	1967	Dibutyl phthalate	E	1.015	0.027	0.006	-	-	RI, GC/MS	-
104	2114	2114	Phytol	O	0.774	0.229	0.036	0.234	0.040	RI, GC/MS	GC/MS
105	-	- ^7^	2.2.4*a*.7*a*-Tetramethyldecahydro-1H-cyclobuta[e]inden-5-ol	SO	-	-	-	0.124	0.028	-	GC/MS
106	-	-	2.5-Anhydro-1-*O*-octylhexitol	O	-	-	-	0.133	0.049	-	GC/MS
			**Chemical classes**								
			Aldehydes			1.338		0.991			
			Ketones			0.053		0.047			
			Fatty acids			-		0.724			
			Esters			0.061		-			
			Monoterpene hydrocarbons			14.683		11.109			
			Oxygenated monoterpenes			20.730		17.447			
			Sesquiterpene hydrocarbons			45.838		54.019			
			Oxygenated sesquiterpenes			12.639		11.972			
			Others			0.229		0.367			
			Total identified (%)			95.571		96.676			

^1^ Retention indices: Obs = retention indices determined relative to a homologous series of *n*-alkanes (C8-C40) on a HP-5MS column, Lit = literature RI values [32,33]; ^2^ C = chemical class: A—aldehydes, E—esters, FA—fatty acid, K—ketones, MH—monoterpene hydrocarbons, MO—oxygenated monoterpenes, O—others, SH—sesquiterpene hydrocarbons, SO—oxygenated sesquiterpenes; ^3^ RF = response factor; ^4^ column = composition of essential oil detected on HP-5MS and DB-HeavyWAX columns; (%) = relative percentage content; c = content is expressed as concentration in milligram per 1 kg of dry plant material; ^5^ identification method: GC/MS = mass spectrum was identical to that of the National Institute of Standards and Technology Library (ver. 2.0.f), RI = the retention index was matching literature database; Std = constituent identity confirmed by coinjection of authentic standards; ^6^ retention indices were not calculated for compounds identified only by DB-HeavyWAX column; ^7^ literature data not available; ^8^ not detected.

**Table 6:** Chemical composition of *Xanthostemon verdugonianus* leaf essential oil.

	RI ^1^		Compound	C ^2^	RF ^3^	Column ^4^				Identification ^5^	
	Obs.	Lit.				HP-5MS		DB-HeavyWAX		HP-5MS	DB-HeavyWAX
						(%)	c	(%)	c		
1	1341	1335	γ-Elemen	SH	0.751	0.062	0.007	- ^8^	-	RI, GC/MS	-
2	1377	1374	Isoledene	SH	0.751	0.063	0.007	-	-	RI, GC/MS	-
3	1388	1389	β-Elemen	SH	0.751	3.015	0.350	1.999	0.377	RI, GC/MS	GC/MS
4	1419	1409	α-Gurjunene	SH	0.751	32.285	3.741	19.519	3.648	RI, GC/MS	GC/MS
5	1425	1418	Caryophyllene	SH	0.751	6.386	0.739	2.987	0.559	RI, GC/MS, Std	-
6	1444	1443	Aromandendrene	SH	0.751	0.263	0.035	0.195	0.037	RI, GC/MS	GC/MS
7	1455	1479	γ-Himachalene	SH	0.751	0.063	0.007	0.060	0.011	RI, GC/MS	GC/MS
8	1460	1452	Humulene	SH	0.751	0.724	0.082	0.417	0.079	RI, GC/MS, Std	GC/MS
9	1478	1477	γ-Gurjunene	SH	0.751	2.097	0.242	1.028	0.194	RI, GC/MS	GC/MS
10	1480	1479	γ-Selinene	SH	0.751	0.289	0.034	0.128	0.024	RI, GC/MS	GC/MS
11	1486	1484	Isogermacrene D	SH	0.751	0.071	8000	**-**	-	RI, GC/MS	-
12	1492	1489	β-Eudesmene	SH	0.751	0.235	0.027	0.148	0.028	RI, GC/MS	GC/MS
13	1503	1494	β-Cyclogermacrane	SH	0.751	5.250	0.592	1.898	0.358	RI, GC/MS	GC/MS
14	- ^6^	1496	Viridiflorene	SH	-	-	-	0.846	0.166	-	GC/MS
15	-	1498	α-Selinene	SH	-	-	-	0.134	0.025	-	GC/MS
16	1519	1513	γ-Cadinene	SH	0.751	0.398	0.046	2.611	0.488	RI, GC/MS	GC/MS
17	1530	1522	δ-Cadinene	SH	0.751	2.463	0.280	-	-	RI, GC/MS	-
18	-	1522	Calamenene	SH	-	-	-	0.058	0.010	-	GC/MS
19	1538	1535	Cubenene	SH	0.751	0.047	0.005	-	-	RI, GC/MS	-
20	1544	1537	α-Cadinene	SH	0.751	0.107	0.012	0.254	0.048	RI, GC/MS	GC/MS
21	-	1541	α-Copaen-11-ol	SO	-	-	-	0.914	0.191	-	GC/MS
22	1577	1567	Palustrol	SO	0.819	1.930	0.238	1.123	0.231	RI, GC/MS	GC/MS
23	-	1567	Maaliol	SO	-	-	-	0.091	0.019	-	GC/MS
24	1586	1576	Spathulenol	SO	0.830	0.582	0.075	0.428	0.089	RI, GC/MS	GC/MS
25	-	1583	Caryophyllene oxide	SH	-	-	-	0.089	0.019	-	GC/MS
26	1593	1590	Globulol	SO	0.819	1.297	0.160	0.624	0.123	RI, GC/MS	GC/MS
27	-	1595	Cubeban-11-ol	SO	-	-	-	0.267	0.055	-	GC/MS
28	1601	1600	Viridiflorol	SO	0.819	1.017	0.125	0.493	0.092	RI, GC/MS	GC/MS
29	-	1600	Rosifoliol	SO	-	-	-	0.100	0.020	-	GC/MS
30	1614	1602	Ledol	SO	0.819	3.629	0.445	1.517	0.312	RI, GC/MS	GC/MS
31	-	1619	1,10-di-epi-Cubenol	SO	-	-	-	0.074	0.015	-	GC/MS
32	1622	1630	α-Acorenol	SO	0.819	0.108	0.012	-	-	RI, GC/MS	-
33	1634	1755	α-Vetivol	SO	0.830	0.781	0.079	0.734	0.153	RI, GC/MS	GC/MS
34	1651	1640	α-epi-Muurolol	SO	0.819	2.644	0.334	0.612	0.126	RI, GC/MS	GC/MS
35	1655	1645	δ-Cadinol	SO	0.819	0.397	0.047	-	-	RI, GC/MS	-
36	-	1650	β-Eudesmol	SO	-	-	-	0.138	0.029	-	GC/MS
37	1661	- ^7^	1-(3-Methyl-2-cyclopenten-1-yl)-1-cyclohexene	O	0.765	1.023	0.118	-	-	GC/MS	-
38	1665	1652	α-Cadinol	SO	0.819	3.730	0.473	2.628	0.534	RI, GC/MS	GC/MS
39	-	1662	Longifolenaldehyde	SO	-	-	-	0.444	0.093	-	GC/MS
40	1684	1678	Alloaromadendrene oxide-(2)	SO	0.830	0.062	0.008	-	-	RI, GC/MS	-
41	1704	-	γ-Gurjunenepoxide-(2)	SO	0.830	0.152	0.019	-	-	GC/MS	-
42	1710	1711	Valerenol	SO	0.830	0.214	0.027	0.067	0.014	RI, GC/MS	GC/MS
43	1724	-	6-Isopropenyl-4,8a-dimethyl-1,2,3,5,6,7,8,8a-octahydro-2-naphthalenol	SO	0.830	0.318	0.042	**-**	-	GC/MS	-
44	1746	1723	Isolongifolen-9-one	SO	0.841	0.077	0.010	-	-	RI, GC/MS	-
45	1754	1730	2,2,7,7-Tetramethyltricyclo[6.2.1.0(1,6)]undec-4-en-3-one	SO	0.841	0.057	0.008	0.078	0.017	RI, GC/MS	GC/MS
46	1765	1766	Costol	SO	0.830	0.118	0.016	0.148	0.031	RI, GC/MS	GC/MS
47	1785	1717	Cyperenone	SO	0.841	22.653	2.745	52.694	10.958	RI, GC/MS	GC/MS
48	1835	-	Spiro[tricyclo[4.4.0.0(5,9)]decane-10,2′-oxirane], 1-methyl-4-isopropyl-7,8-dihydroxy-, (8S)-	O	0.999	0.073	0.011	0.081	0.012	GC/MS	GC/MS
49	1910	-	6-[1-(Hydroxymethyl)vinyl]-4,8a-dimethyl-3,5,6,7,8,8a-hexahydro-2(1H)-naphthalenone	SO	0.926	0.077	0.011	-	-	GC/MS	-
50	-	-	Neointermedeol	SO	-	-	-	0.423	0.087	-	GC/MS
51	-	-	Tricyclo[5.3.1.1(2,6)]dodecan-11-ol, 11-methyl-12-methylene-	MO	-	-	-	0.065	0.014	-	GC/MS
			**Chemical classes**								
			Oxygenated monoterpenes			-		0.065			
			Sesquiterpenes hydrocarbons			53.818		32.371			
			Oxygenated sesquiterpenes			39.843		63.597			
			Others			1.096		0.081			
			Total identified (%)			94.757		96.114			

^1^ Retention indices: Obs = retention indices determined relative to a homologous series of *n*-alkanes (C8-C40) on a HP-5MS column, Lit = literature RI values [32,33]; ^2^ C = chemical class: MO—oxygenated monoterpenes, O—others, SH—sesquiterpene hydrocarbons, SO—oxygenated sesquiterpenes; ^3^ RF = response factor; ^4^ column = composition of essential oil detected on HP-5MS and DB-HeavyWAX columns; (%) = relative percentage content; c = content is expressed as concentration in milligram per 1 kg of dry plant material; ^5^ identification method: GC/MS = Mass spectrum was identical to that of the National Institute of Standards and Technology Library (ver. 2.0.f), RI = the retention index was matching literature database; Std = constituent identity confirmed by coinjection of authentic standards; ^6^ retention indices were not calculated for compounds identified only by DB-HeavyWAX column; ^7^ literature data not available; ^8^ not detected.

**Table 7:** Chemical composition of *Nigella sativa* seed supercritical CO_2_ extract.

	RI ^1^		Compound	C ^2^	RF ^3^	Column ^4^				Identification ^5^	
	Obs.	Lit.				HP-5MS		DB-HeavyWAX		HP-5MS	DB-HeavyWAX
						(%)	c	(%)	c		
1	- ^6^	1024	o-Cymene	MH	-	- ^8^	-	0.113	0.010	-	GC/MS
2	1026	1024	D-Limonene	MH	0.765	0.376	0.015	0.089	0.009	RI, GC/MS	GC/MS
3	1115	1120	4-methoxy thujane	MO	0.852	0.130	0.003	0.240	0.028	RI, GC/MS	GC/MS
4	1253	1239	Carvone	MO	0.907	0.473	0.022	-	-	RI, GC/MS, Std	-
5	1262	1248	Thymoquinone	K	1.071	0.700	0.037	-	-	RI, GC/MS	-
6	1298	1282	Anethole	MO	0.824	0.106	0.004	-	-	RI, GC/MS	-
7	1324	1317	2,4-Decadienal	A	0.887	0.080	0.003	0.096	0.012	RI, GC/MS	GC/MS
8	1349	1346	α-Terpinyl acetate	E	0.957	0.204	0.009	-	-	RI, GC/MS	-
9	1419	1418	Caryophyllene	SH	0.751	0.160	0.006	0.066	0.006	RI, GC/MS, Std	GC/MS
10	-	1765	Tetradecanoic acid	FA	-	-	-	0.186	0.021	-	GC/MS
11	1958	1961	Sandaracopimaradiene	DH	0.744	0.119	0.003	-	-	RI, GC/MS	-
12	-	1960	Hexadecanoic acid	FA	-	-	-	9.897	1.097	-	GC/MS
13	1982	1982	Ethyl hexadecanoate	E	0.845	0.205	0.009	0.102	0.011	RI, GC/MS	GC/MS
14	-	2142	Oleic acid	FA	-	-	-	19.576	2.208	-	GC/MS
15	2155	2155	Ethyl linolate	E	0.846	5.023	0.138	1.582	0.174	RI, GC/MS	GC/MS
16	2159	2149	Ethyl oleate	E	0.838	2.782	0.072	0.265	0.030	RI, GC/MS	GC/MS
17	2224	2132	Linoleic acid	FA	0.863	71.657	3.019	59.245	6.713	RI, GC/MS	GC/MS
18	2559	2540	Diisooctyl phthalate	E	0.900	0.334	0.014	-	-	RI, GC/MS	-
19	-	- ^7^	1,2-15,16-Diepoxyhexadecane	O	-	-	-	0.090	0.010	-	GC/MS
20	-	-	β-Monoolein	E	-	-	-	0.761	0.091	-	GC/MS
			**Chemical classes**								
			Aldehydes			0.080		0.096			
			Ketones			0.700		-			
			Fatty acids			71.657		88.904			
			Esters			8.548		2.710			
			Monoterpene hydrocarbons			0.376		0.202			
			Diterpene hydrocarbons			0.119		-			
			Oxygenated monoterpenes			0.709		0.240			
			Sesquiterpene hydrocarbons			0.160		0.066			
			Others			-		0.090			
			Total identified (%)			82.349		92.308			

^1^ Retention indices: Obs = retention indices determined relative to a homologous series of *n*-alkanes (C8-C40) on a HP-5MS column, Lit = literature RI values [32,33]; ^2^ C = chemical class: A—aldehydes, DH—diterpene hydrocarbons, E—esters, FA—fatty acid, K—ketones, MH—monoterpene hydrocarbons, MO—oxygenated monoterpenes, O—others, SH—sesquiterpene hydrocarbons; ^3^ RF = response factor; ^4^ column = composition of essential oil detected on HP-5MS and DB-HeavyWAX columns; (%) = relative percentage content; c = content is expressed as concentration in milligram per 1 kg of dry plant material; ^5^ identification method: GC/MS = mass spectrum was identical to that of the National Institute of Standards and Technology Library (ver. 2.0.f), RI = the retention index was matching literature database; Std = constituent identity confirmed by coinjection of authentic standards; ^6^ retention indices were not calculated for compounds identified only by DB-HeavyWAX column; ^7^ literature data not available; ^8^ not detected.

**Table 8:** Botanical description and physicochemical characteristic of plant species and samples tested.

Scientific Name	Family	Voucher Specimen/Sample Number	Area of Collection	Part Used	Isolation Technique	Yield %	Color
*Alpinia elegans* (C.Presl) K.Schum.	Zingiberaceae	02509KBFR7	Mt Pangasugan, PHL	Seed	HD ^1^	0.52	Yellow
*Cinnamomum iners* Reinw. ex Blume	Lauraceae	02577KBFRC	Mt Pangasugan, PHL	Leaf	HD	0.52	Pale yellow
*Nigella sativa* L.	Ranunculaceae	02604KBFR3	U Salvatora, Prague, CZ	Seed	SFE ^2^	5.80	Pale greenish yellow
*Xanthostemon verdugonianus* Náves ex Fern.-Vill.	Myrtaceae	02581KBFR7	Mt Pangasugan, PHL	Leaf	HD	2.86	Pale yellow

^1^ HD: hydrodistillation, ^2^ SFE: supercritical fluid extraction.

## References

[1] Bennett J.W., Inamdar A.A. (2015). Are some fungal volatile organic compounds (VOCs) mycotoxins?. Toxins.

[2] Altındal D., Altındal N., Choudhary D.K., Sharma A.K., Agarwal P., Varma A., Tuteja N. (2017). Plant volatile compounds in growth. Volatiles and Food Security.

[3] Margetts J., Rowe D.J. (2005). Aroma chemicals V: Natural aroma chemicals. Chemistry and Technology of Flavors and Fragrances.

[4] Kumari S., Pundhir S., Priya P., Jeena G., Punetha A., Chawla K., Jafaree Z.F., Mondal S., Yadav G. (2014). EssOilDB: A database of essential oils reflecting terpene composition and variability in the plant kingdom. Database.

[5] Damasceno C.S.B., Higaki N.T.F., Dias J.D.G., Miguel M.D., Miguel O.G. (2019). Chemical composition and biological activities of essential oils in the family Lauraceae: A systematic review of the literature. Planta Med..

[6] Farias D.P., Neri-Numa I.A., de Araujo F.F., Pastore G.M. (2020). A critical review of some fruit trees from the Myrtaceae family as promising sources for food applications with functional claims. Food Chem..

[7] Gilli C., He Z., But P.P., Schinnerl J., Valant V.K.M., Greger H. (2011). Chemodiversity and biological activity of the genus *Alpinia* (Zingiberaceae). Planta Med..

[8] Baptista-Silva S., Borges S., Ramos O.L., Pintado M., Sarmento B. (2020). The progress of essential oils as potential therapeutic agents: A review. J. Essent. Oil Res..

[9] Islam M.T., Khan M., Mishra S.K. (2019). An updated literature-based review: Phytochemistry, pharmacology and therapeutic promises of *Nigella sativa* L. Orient. Pharm. Exp. Med..

[10] Ghahramanloo K.H., Kamalidehghan B., Javar H.A., Widodo R.T., Majidzadeh K., Noordin M.I. (2017). Comparative analysis of essential oil composition of Iranian and Indian *Nigella sativa* L. extracted using supercritical fluid extraction and solvent extraction. Drug Des. Dev. Ther..

[11] Shaaban H.A.E., El-Ghorab A.H., Shibamoto T. (2012). Bioactivity of essential oils and their volatile aroma components: Review. J. Essent. Oil Res..

[12] Kokoska L., Kloucek P., Leuner O., Novy P. (2019). Plant-derived products as antibacterial and antifungal agents in human health care. Curr. Med. Chem..

[13] Guzman G.Q., Dacanay A.T.L., Andaya B.A., Alejandro G.J.D. (2016). Ethnopharmacological studies on the uses of *Euphorbia hirta* in the treatment of dengue in selected indigenous communities in Pangasinan (Philippines). J. Intercult. Ethnopharmacol..

[14] Jachak S.M., Saklani A. (2007). Challenges and opportunities in drug discovery from plants. Curr. Sci..

[15] Kavanagh A., Ramu S., Gong Y., Cooper M.A., Blaskovich M.A.T. (2019). Effects of microplate type and broth additives on microdilution MIC susceptibility assays. Antimicrob. Agents Chemother..

[16] Casey J.T., O’Cleirigh C., Walsh P.K., O’Shea D.G. (2004). Development of a robust microtiter plate-based assay method for assessment of bioactivity. J. Microbiol. Methods.

[17] Balouiri M., Sadiki M., Ibnsouda S.K. (2016). Methods for in vitro evaluating antimicrobial activity: A review. J. Pharm. Anal..

[18] Bobo-Garcia G., Davidov-Pardo G., Arroqui C., Virseda P., Marin-Arroyo M.R., Navarro M. (2015). Intra-laboratory validation of microplate methods for total phenolic content and antioxidant activity on polyphenolic extracts, and comparison with conventional spectrophotometric methods. J. Sci. Food Agric..

[19] Martin A., Clynes M. (1993). Comparison of 5 microplate colorimetric assays for in vitro cytotoxicity testing and cell proliferation assays. Cytotechnology.

[20] Thielmann J., Muranyi P., Kazman P. (2019). Screening essential oils for their antimicrobial activities against the foodborne pathogenic bacteria *Escherichia coli* and *Staphylococcus aureus*. Heliyon.

[21] Lin C.W., Yu C.W., Wu S.C., Yih K.H. (2009). DPPH free-radical scavenging activity, total phenolic contents and chemical composition analysis of forty-two kinds of essential oils. J. Food Drug Anal..

[22] Al-Tamimi M.A., Rastall B., Abu-Reidah I.M. (2016). Chemical composition, cytotoxic, apoptotic and antioxidant activities of main commercial essential oils in Palestine: A comparative study. Medicines.

[23] Houdkova M., Kokoska L. (2020). Volatile antimicrobial agents and in vitro methods for evaluating their activity in the vapour phase: A review. Planta Med..

[24] Reyes-Jurado F., Franco-Vega A., Ramirez-Corona N., Palou E., Lopez-Malo A. (2015). Essential oils: Antimicrobial activities, extraction methods and their modeling. Food Eng. Rev..

[25] Kalemba D., Kunicka A. (2003). Antibacterial and antifungal properties of essential oils. Curr. Med. Chem..

[26] Fisher K., Phillips C. (2008). Potential antimicrobial uses of essential oils in food: Is citrus the answer?. Trends Food Sci. Technol..

[27] Novy P., Kloucek P., Rondevaldova J., Havlik J., Kourimska L., Kokoska L. (2014). Thymoquinone vapour significantly affects the results of *Staphylococcus aureus* sensitivity tests using the standard broth microdilution method. Fitoterapia.

[28] Rondevaldova J., Novy P., Urban J., Kokoska L. (2017). Determination of anti-staphylococcal activity of thymoquinone in combinations with antibiotics by checkerboard method using EVA capmat (TM) as a vapor barrier. Arab. J. Chem..

[29] Houdkova M., Rondevaldova J., Doskocil I., Kokoska L. (2017). Evaluation of antibacterial potential and toxicity of plant volatile compounds using new broth microdilution volatilization method and modified MTT assay. Fitoterapia.

[30] Karki N., Aggarwal S., Laine R.A., Greenway F., Losso J.N. (2020). Cytotoxicity of juglone and thymoquinone against pancreatic cancer cells. Chem. Biol. Interact..

[31] Chraibi M., Farah A., Elamin O., Iraqui H.I., Fikri-Benbrahim K. (2020). Characterization, antioxidant, antimycobacterial, antimicrobial effcts of Moroccan rosemary essential oil, and its synergistic antimicrobial potential with carvacrol. J. Adv. Pharm. Technol. Res..

[32] NIST WebBook Chemie (2017). NIST Standard Reference Database Number 69. http://webbook.nist.gov/chemistry/.

[33] Adams R.P. (2007). Identification of Essential Oil Components by Gas Chromatography/Mass Spectrometry.

[34] Thellen C., Blaise C., Roy Y., Hickey C. (1989). Round Robin testing with the *Selenastrum capricornutum* microplate toxicity assay. Hydrobiologia.

[35] Feyaerts A.F., Mathe L., Luyten W., Tournu H., Van Dyck K., Broekx L., Van Dijck P. (2017). Assay and recommendations for the detection of vapour-phase-mediated antimicrobial activities. Flavour Fragr. J..

[36] Van Vuuren S.F., Kamatou G.P.P., Viljoen A.M. (2010). Volatile composition and antimicrobial activity of twenty commercial frankincense essential oil samples. S. Afr. J. Bot..

[37] Piras A., Rosa A., Marongiu B., Porcedda S., Falconieri D., Dessi M.A., Ozcelik B., Koca U. (2013). Chemical composition and in vitro bioactivity of the volatile and fixed oils of *Nigella sativa* L. extracted by supercritical carbon dioxide. Ind. Crops Prod..

[38] Houdkova M., Doskocil I., Urbanova K., Tulin E.K.C.B., Rondevaldova J., Tulin A.B., Kudera T., Tulin E.E., Zeleny V., Kokoska L. (2018). Evaluation of antipneumonic effect of Philippine essential oils using broth microdilution volatilization method and their lung fibroblasts toxicity. Nat. Prod. Commun..

[39] Mustaffa F., Indurkar J., Ismail S., Shah M., Mansor S.M. (2011). An antimicrobial compound isolated from *Cinnamomum iners* leaves with activity against methicillin-resistant *Staphylococcus aureus*. Molecules.

[40] Visutthi M. (2016). Anti-staphylococcal screening of selected Thai medicinal plants from Nakhon Ratchasima province. Suranaree J. Sci. Technol..

[41] Wang Z.C., Wei B.Y., Pei F.N., Yang T., Tang J., Yang S., Yu L.F., Yang C.G., Yang F. (2020). Capsaicin derivatives with nitrothiophene substituents: Design, synthesis and antibacterial activity against multidrug-resistant *S. aureus*. Eur. J. Med. Chem..

[42] Schmidt E., Bail S., Friedl S.M., Jirovetz L., Buchbauer G., Wanner J., Denkova Z., Slavchev A., Stoyanova A., Geissler M. (2010). Antimicrobial activities of single aroma compounds. Nat. Prod. Commun..

[43] Srisung S., Suksrichavalit T., Prachayasittikul S., Ruchirawat S., Prachayasittikul V. (2013). Antimicrobial activity of 8-hydroxyquinoline and transition metal complexes. Int. J. Pharmacol..

[44] Hariharan P., Paul-Satyaseela M., Gnanamani A. (2016). In vitro profiling of antimethicillin-resistant *Staphylococcus aureus* activity of thymoquinone against selected type and clinical strains. Lett. Appl. Microbiol..

[45] Phutdhawong W., Kawaree R., Sanjaiya S., Sengpracha W., Buddhasukh D. (2007). Microwave-assisted isolation of essential oil of *Cinnamomum iners* Reinw. ex Bl: Comparison with conventional hydrodistillation. Molecules.

[46] Naive M.A.K., Dalisay J.A.G.P., Maglangit E.P.T., Cafe G.C., Nuneza O.M. (2019). Free radical scavenging effects of the Philippine endemic medicinal plant *Alpinia elegans* (Zingiberaceae). Gard. Bull. Singap..

[47] Solati Z., Baharin B.S., Bagheri H. (2014). Antioxidant property, thymoquinone content and chemical characteristics of different extracts from *Nigella sativa* L. seeds. J. Am. Oil Chem. Soc..

[48] Karakaya S., Yilmaz S.V., Ozdemir O., Koca M., Pinar N.M., Demirci B., Yildirim K., Sytar O., Turkez H., Baser K.H.C. (2020). A caryophyllene oxide and other potential anticholinesterase and anticancer agent in *Salvia verticillata* subsp. amasiaca (Freyn & Bornm.) Bornm. (Lamiaceae). J. Essent. Oil Res..

[49] Nascimento P.L.A., Nascimento T.C.E.S., Ramos S.M., Silva G.R., Gomes J.E.G., Falcao R.E.A., Moreira K.A., Porto A.L.F., Silva T.M.S. (2014). Quantification, antioxidant and antimicrobial activity of phenolics isolated from different extracts of *Capsicum frutescens* (Pimenta Malagueta). Molecules.

[50] Cherdtrakulkiat R., Boonpangrak S., Sinthupoom N., Prachayasittikul S., Ruchirawat S., Prachayasittikul V. (2016). Derivatives (halogen, nitro and amino) of 8-hydroxyquinoline with highly potent antimicrobial and antioxidant activities. Biochem. Biophys. Rep..

[51] Yildiz S., Turan S., Kiralan M., Ramadan M.F. (2020). Antioxidant properties of thymol, carvacrol, and thymoquinone and its efficiencies on the stabilization of refined and stripped corn oils. J. Food Meas. Charact..

[52] Mustafa F., Indurkar J., Ismail S., Mordi M.N., Ramanathan S., Mansor S.M. (2010). Antioxidant capacity and toxicity screening of *Cinnamomum iners* standardized leaves methanolic extract. Int. J. Pharmacol..

[53] Baharetha H.M., Nassar Z.D., Aisha A.F., Ahamed M.B.K., Al-Suede F.S.R., Kadir M.O.A., Ismail Z., Majid A.M.S.A. (2013). Proapoptotic and antimetastatic properties of supercritical CO_2_ extract of *Nigella sativa* Linn. against breast cancer cells. J. Med. Food.

[54] Richeux F., Cascante M., Ennamany R., Saboureau D., Creppy E.E. (1999). Cytotoxicity and genotoxicity of capsaicin in human neuroblastoma cells SHSY-5Y. Arch. Toxicol..

[55] Ambroz M., Bousova I., Skarka A., Hanusova V., Kralova V., Matouskova P., Szotakova B., Skalova L. (2015). The influence of sesquiterpenes from Myrica rubra on the antiproliferative and pro-oxidative effects of doxorubicin and its accumulation in cancer cells. Molecules.

[56] Reis D.C., Pinto M.C.X., Souza-Fagundez E.M., Rocha L.F., Pereira V.R.A., Melo C.M.L., Beraldo H. (2011). Investigation on the pharmacological profile of antimony(III) complexes with hydroxyquinoline derivatives: Anti-trypanosomal activity and cytotoxicity against human leukemia cell lines. Biometals.

[57] Norsharina I., Maznah I., Aied A.A., Ghanya A.N. (2011). Thymoquinone rich fraction from *Nigella sativa* and thymoquinone are cytotoxic towards colon and leukemic carcinoma cell lines. J. Med. Plant Res..

[58] Son L.C., Dai D.N., Thai T.H., Huyen D.D., Thang T.D., Ogunwande I.A. (2013). The leaf essential oils of four Vietnamese species of *Cinnamomum* (Lauraceae). J. Essent. Oil Res..

[59] Venkatachallam S.K.T., Pattekhan H., Divakar S., Kadimi U.S. (2010). Chemical composition of *Nigella sativa* L. seed extracts obtained by supercritical carbon dioxide. J. Food Sci. Technol..

[60] Machmudah S., Shiramizu Y., Goto M., Sasaki M., Hirose T. (2005). Extraction of *Nigella sativa* L. using supercritical CO_2_: A study of antioxidant activity of the extract. Sep. Sci. Technol..

[61] Tissot E., Rochat S., Debonneville C., Chaintreau A. (2012). Rapid GC-FID quantification technique without authentic samples using predicted response factors. Flavour Fragr. J..

[62] European Directorate for the Quality of Medicines and Healthcare (EDQM) (2014). Essential oils in herbal drugs (Monograph 2. 8.12.). European Pharmacopoeia.

[63] Clinical and Laboratory Standards Institute (CLSI) (2012). Methods for Dilution Antimicrobial Susceptibility Tests for Bacteria That Grow Aerobically; Approved Standard.

[64] Clinical and Laboratory Standards Institute (CLSI) (2015). Performance Standards for Antimicrobial Susceptibility Testing.

[65] Sharma O.P., Bhat T.K. (2009). DPPH antioxidant assay revisited. Food Chem..

[66] Mosmann T. (1983). Rapid colorimetric assay for cellular growth and survival: Application to proliferation and cytotoxicity assays. J. Immunol. Methods.

[67] Special Programme for Research and Training in Tropical Diseases. http://www.who.int/tdr/grants/workplans/en/cytotoxicity_invitro.pdf/.

[68] Cachet T., Brevard H., Chaintreau A., Demyttenaere J., French L., Gassenmeier K., Joulain D., Koenig T., Leijs H., Liddle P. (2016). IOFI recommended practice for the use of predicted relative-response factors for the rapid quantification of volatile flavouring compounds by GC-FID. Flavour Fragr. J..

